# Telomere-to-telomere assembly of cassava genome reveals the evolution of cassava and divergence of allelic expression

**DOI:** 10.1093/hr/uhad200

**Published:** 2023-10-05

**Authors:** Xin-Dong Xu, Ru-Peng Zhao, Liang Xiao, Liuying Lu, Min Gao, Yu-Hong Luo, Zu-Wen Zhou, Si-Ying Ye, Yong-Qing Qian, Bing-Liang Fan, Xiaohong Shang, Pingli Shi, Wendan Zeng, Sheng Cao, Zhengdan Wu, Huabing Yan, Ling-Ling Chen, Jia-Ming Song

**Affiliations:** State Key Laboratory for Conservation and Utilization of Subtropical Agro-bioresources, College of Life Science and Technology, Guangxi University, Nanning 530004, China; State Key Laboratory for Conservation and Utilization of Subtropical Agro-bioresources, College of Life Science and Technology, Guangxi University, Nanning 530004, China; Cash Crops Research Institute, Guangxi Academy of Agricultural Sciences, Nanning 530007, China; Cash Crops Research Institute, Guangxi Academy of Agricultural Sciences, Nanning 530007, China; State Key Laboratory for Conservation and Utilization of Subtropical Agro-bioresources, College of Life Science and Technology, Guangxi University, Nanning 530004, China; State Key Laboratory for Conservation and Utilization of Subtropical Agro-bioresources, College of Life Science and Technology, Guangxi University, Nanning 530004, China; State Key Laboratory for Conservation and Utilization of Subtropical Agro-bioresources, College of Life Science and Technology, Guangxi University, Nanning 530004, China; State Key Laboratory for Conservation and Utilization of Subtropical Agro-bioresources, College of Life Science and Technology, Guangxi University, Nanning 530004, China; State Key Laboratory for Conservation and Utilization of Subtropical Agro-bioresources, College of Life Science and Technology, Guangxi University, Nanning 530004, China; State Key Laboratory for Conservation and Utilization of Subtropical Agro-bioresources, College of Life Science and Technology, Guangxi University, Nanning 530004, China; Cash Crops Research Institute, Guangxi Academy of Agricultural Sciences, Nanning 530007, China; Cash Crops Research Institute, Guangxi Academy of Agricultural Sciences, Nanning 530007, China; Cash Crops Research Institute, Guangxi Academy of Agricultural Sciences, Nanning 530007, China; Cash Crops Research Institute, Guangxi Academy of Agricultural Sciences, Nanning 530007, China; Cash Crops Research Institute, Guangxi Academy of Agricultural Sciences, Nanning 530007, China; Cash Crops Research Institute, Guangxi Academy of Agricultural Sciences, Nanning 530007, China; State Key Laboratory for Conservation and Utilization of Subtropical Agro-bioresources, College of Life Science and Technology, Guangxi University, Nanning 530004, China; State Key Laboratory for Conservation and Utilization of Subtropical Agro-bioresources, College of Life Science and Technology, Guangxi University, Nanning 530004, China

## Abstract

Cassava is a crucial crop that makes a significant contribution to ensuring human food security. However, high-quality telomere-to-telomere cassava genomes have not been available up to now, which has restricted the progress of haploid molecular breeding for cassava. In this study, we constructed two nearly complete haploid resolved genomes and an integrated, telomere-to-telomere gap-free reference genome of an excellent cassava variety, ‘Xinxuan 048’, thereby providing a new high-quality genomic resource. Furthermore, the evolutionary history of several species within the Euphorbiaceae family was revealed. Through comparative analysis of haploid genomes, it was found that two haploid genomes had extensive differences in linear structure, transcriptome features, and epigenetic characteristics. Genes located within the highly divergent regions and differentially expressed alleles are enriched in the functions of auxin response and the starch synthesis pathway. The high heterozygosity of cassava ‘Xinxuan 048’ leads to rapid trait segregation in the first selfed generation. This study provides a theoretical basis and genomic resource for molecular breeding of cassava haploids.

## Introduction

Cassava (*Manihot esculenta*) (2*n* = 36) is a staple crop extensively cultivated in tropical areas, serving as a vital source of sustenance for over one billion individuals inhabiting the African, South American, and Asian tropical regions [1]. Despite its significance, identifying allelic variations and functional differences of divergent alleles in the highly heterozygous cassava genome is challenging [2, 3]. Unfortunately, due to lack of high-quality reference genomes [4], studies on cassava genomics and breeding have lagged. The availability of such a reference genome would provide an invaluable resource for the development of superior cassava varieties exhibiting increased yield under biotic and abiotic stresses. Moreover, it would also facilitate evolutionary studies to enhance our understanding of this vital crop.

With the ongoing development of sequencing technology and assembly algorithms, the genomes of seven cassava accessions, AM560-2, W14, KU50, TME3, 60 444, SC205, and TME204, have been sequenced [3, 5–7]. However, most of them are low-quality draft genomes, and there are still challenges in cassava genomic research, such as the identification of centromere regions, the evaluation of evolutionary trajectories, and discrepancies in haplotype variation, alleles, and epigenetics [8, 9]. Telomere-to-telomere (T2T) genome assemblies have been performed on a variety of species, resolving numerous biological investigations. For example, T2T assemblies of human [10], *Arabidopsis* [11], and rice [9] genomes have clarified the evolution of their centromeres. T2T genome assembly of mulberry [12] has revealed its multiple-centromere phenomenon and proposed a model of the chromosomal fission–fusion cycle in mulberry. Furthermore, T2T genome assemblies of *Saccharum* [13] and strawberry [14] have revealed their intricate evolutionary histories. These fundamental studies provide the basis for T2T genome assembly of cassava.

‘Xinxuan 048’ (XX048) is a representative cassava variety, which exhibits remarkable qualities, such as strong growth, high productivity, and excellent stress resistance [15] (Supplementary Data Fig. S1). Furthermore, the first selfed generation (*S*_1_) of XX048 showed diversified genetic variants differing in plant height, leaflet count, leaflet length, underground weight, and so on (Fig. 5a–c, Supplementary Data Fig. S14, Supplementary Data Table S11). To elucidate the profound mystery of this phenomenon, two nearly complete haplotype-resolved assemblies of XX048 were obtained by using PacBio high-fidelity (HiFi) sequencing and high-throughput chromosome conformation capture (Hi-C) sequencing in this study. Following this, gaps in the genome were successfully filled by utilizing Oxford Nanopore Technologies (ONT) reads from self-crossed progeny of cassava XX048, leading to the first T2T cassava genome. Furthermore, the evolutionary process of the cassava karyotype was analyzed. Collinearity within haplotypes and pan-genomic analyses among different varieties were conducted to characterize the diversity of cassava. Analysis of the transcriptome and epigenetics of XX048 revealed disparities in allele expression and methylation levels between the two haploid genomes. To conclude, the T2T genome of XX048 has increased the genomic resources of cassava, allowing us to analyze the evolutionary process of cassava and the disparity between haploids, thus providing a theoretical basis for cassava breeding.

## Results

### Genome assembly, annotation, and centromere prediction

We initially sequenced the genome of cassava variety XX048, generating 38 Gb (~55× coverage and ~15 kb N50) PacBio HiFi reads, 58 Gb (~170× coverage) Hi-C paired reads, 23 Gb (~35× coverage and ~26 kb N50) ONT reads, and 84 Gb (~249× coverage) Illumina PCR free short reads (Supplementary Data Table S1). Employing the *k*-mer method, the estimated genome size of XX048 is 687 Mb, and the genome heterozygosity is 0.7–0.9% (Supplementary Data Fig. S2a and b), which are similar to the size and heterozygosity of other cassava genomes [3, 6]. Due to the high heterozygosity of the XX048 genome, it could be constructed as diploid contigs using PacBio HiFi reads, and two sets of haplotype-resolved genomes were created based on Hi-C paired reads. Subsequently, Hi-C reads were used to scaffold contigs. More than 99% contigs were anchored to XX048 pseudochromosomes. Afterward, iterative and manual adjustments were implemented, including filling gaps and telomere repair to complete the genome assembly (Supplementary Data Fig. S2c).

**Table 1:** Assessment and comparative analysis of XX048 haploid genomes with other cassava genomes.

**Assembly**	**Contig (gap) number**	**Assembly length (Mb)**	**Contig N50 (Mb)**	**BUSCO (C/D%)**	**LAI**	**Telomere number**	**Gap-free number**	**Anchored (%)**	**QV**
XX048 gap-free	18 (0)	664.5	36.5	99.0/8.2	22.73	28	18	100.00	45.587
XX048-hapA	62 (24)	665.2	33.7	99.0/8.0	22.8	28	5	99.17	52.183
XX048-hapB	49 (18)	664.9	32.6	99.0/8.0	21.26	28	6	99.57	52.6661
TME204-hap1 [6]	1439 (25)	762.4	18.4	99.0/8.8	17.46	26	5	86.83	45.4962
TME204-hap2 [6]	770 (21)	706.3	26	98.8/8.0	19	21	5	91.09	49.2458
SC205 [3]	2289 (989)	710	1.1	95.1/16.3	17.73	11	0	90.02	29.5741
AM560 [17]	758 (515)	640.4	3.3	98.9/8.1	21.62	29	0	98.98	45.2119

**Figure 1:**
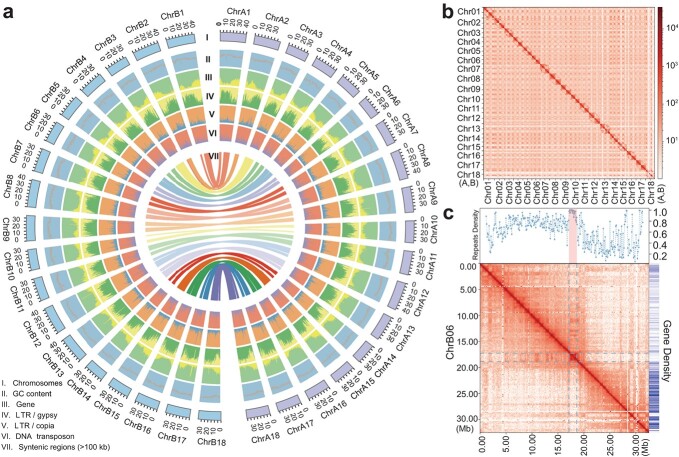
Features of the *M. esculenta* XX048 genome. **a** Circos plot of the *M. esculenta* XX048 genome. (I–VII) From outermost to innermost, concentric circles show chromosomes (I), GC content (II), gene density (III), LTR/*Gypsy* density (IV), LTR/*Copia* density (V), DNA transposon density (VI), and syntenic regions >100 kb between the A and B haplotype genomes (VII). **b** Genome-wide contact matrix of XX048 genome. Color intensity represents the frequency of contact between two 500-kb loci. **c** Candidate centromere region of XX048 ChrB06. The chromatin interaction map, repeat densities, and gene densities are illustrated along the chromosome.

We evaluated and compared the integrated T2T genome and the two haplotype-resolved genomes for XX048 with the recently published manioc genome and found that XX048 genomes are currently the most contiguous, complete, and accurate cassava genomes (Table 1). All 36 chromosomes of diploid cassava were assembled, and after gap-filling with ONT reads an integrated gap-free genome was obtained (Table 1). A total of 56 telomere sequences were identified, and the assembled telomeres had an average length >10 kb. In addition, potential centromere locations were also predicted (Supplementary Data Table S2, Supplementary Data Fig. S3). The assessment of BUSCO single-copy homologous genes indicated assembly completeness of 99.0%, and the long terminal repeat assembly index (LAI) [16] of the two haploid assemblies and integrated gap-free assembly were both >20, meeting the gold standard (Table 1). XX048 haplotype-resolved genomes have the highest quality value (QV) compared with other cassava genomes, being the sole assembly with a QV of ≥50 (or no more than 1 base call error per 100 000 bp) (Table 1). In comparison with the size disparity between the two haplotype-resolved genomes of TME204, the size difference between our two haplotype-resolved genomes is <1 Mb (Table 1), indicating a higher accuracy for our haplotype typing. Analysis of Hi-C data for each chromosome in the XX048 haplotype-resolved genomes reveals strong signals along the main diagonal, indicating frequent interactions between adjacent loci on the chromosome. Notably, significant interactions between homologous chromosomes have also been observed, evidenced by the concentration of Hi-C signals at homologous chromosome boundaries (Fig. 1b). These findings corroborate the correctness of the assembly direction of XX048 assembly and the accuracy of haploid genome typing. By collinearity comparison of XX048 haplotype-resolved genomes with the published AM560 and TME204 genomes, we can see that there is a good collinearity between them (Supplementary Data Fig. S4a–c).

A total of 929 Mb of transposable elements (TEs) were identified in the assembled XX048 haplotype-resolved genomes, representing 69.86% of the genome, a proportion similar to those previously reported in the haploid cassava SC205 [3] and TME204 [6] genomes. Long terminal repeat (LTR) *Gypsy* superfamily TEs had the highest abundance, accounting for 39.5% of the genome, while the *Copia* superfamily TEs had a much lower proportion of only 4.66% of the entire genome. The proportion of retrotransposons (RTs) in the genome was higher than that of DNA transposons (1.32%) (Supplementary Data Table S3). Nevertheless, all three types of transposon had a similar distribution state across both haploid genomes, with most concentrated in regions of low gene density in the middle of the chromosome (Fig. 1a).

After repetitive sequence masking, the genome was used for initial and transcript-evidence-based gene annotation. We integrated protein sequences from three closely related species with transcriptome data of the XX048 variety. We identified a total of 74 980 genes in the two haploid genomes, with an average gene length of 4633 bp and an average coding sequence (CDS) length of 1227 bp (Supplementary Data Table S4). Statistical analyses indicated that the length of genes, CDSs, introns, and exons in the XX048 genome were similar to the previously reported distributions of cassava genomes and related species [17–20] (Supplementary Data Fig. S5), and 96.35% of genes could be annotated by one or more protein databases, suggesting accuracy of the XX048 genome annotation. Additionally, a total of 5950 non-coding RNAs were annotated in the XX048 genome, including 559 microRNAs (miRNAs), 1982 transfer RNAs (tRNAs), 2455 ribosomal RNAs (rRNAs), and 954 small nuclear RNAs (snRNAs) (Supplementary Data Table S5).

The centromere region of the genome is characterized by a high degree of repetitive sequence content, making it a challenging area to assemble [21]. To date, the centromere sequence of the cassava genome has not been analyzed. The nearly complete cassava genome provides the opportunity to investigate this region. Utilizing repeat sequence density distribution, gene quantity density distribution, and Hi-C interaction diagrams, we were able to preliminarily predict the potential location of the centromere of some chromosomes (Supplementary Data Table S2, Fig. 1c, Supplementary Data Fig. S3) and centromere motif sequences and distribution (Supplementary Data Table S6). It was found that multiple chromosomes had the same motif sequence, with the same centromere motif sequence between chromosomes 4 and 15 and between chromosomes 8 and 13 (Supplementary Data Table S6). This indicates that there are different types of centromere motif sequences in the cassava genome, which is different from the single type of centromere motif sequences in the rice [9] and *Arabidopsis* [11] genomes. Upon comparison of the centromeric region of chromosome 8 in XX048 haplotype A with the TME204 haplotype 2 (Supplementary Data Fig. S6), it was observed that the repetitive cycle of centromeric motif sequence of chromosome 8 in XX048 haplotype A had been disrupted by the insertion of TE sequences, suggesting that the insertion of TEs may impede the integrity of the centromere. This is consistent with what has been observed in the barley genome [22].

### Evolutionary history of the cassava genome

**Figure 2:**
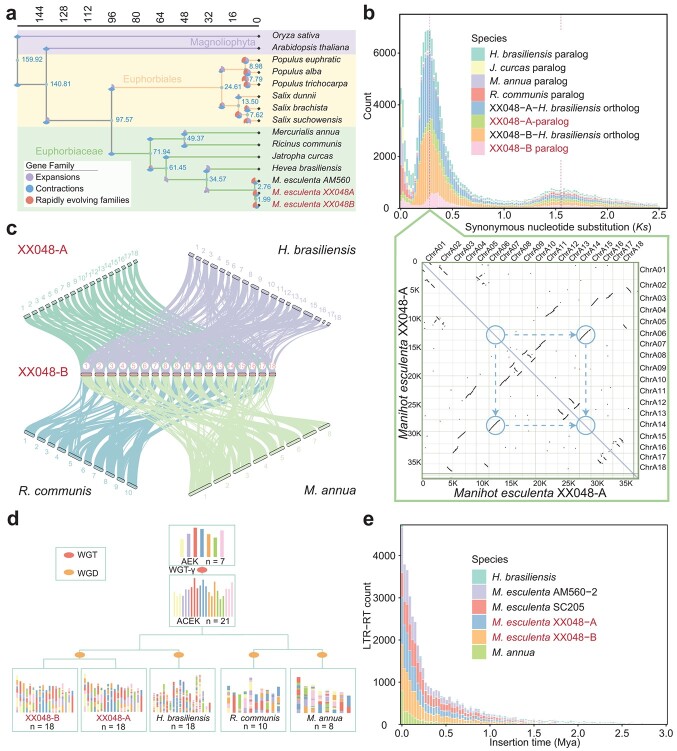
Phylogenetic and comparative genomics analyses of the *M. esculenta* XX048 genome. **a** XX048 haploid genome phylogenetic analysis and divergence time calculation. The tree was built using 776 single-copy, homologous gene families from 15 genomes. **b***K*_s_ distributions and dot plot of paralogs in the XX048 haplotype A genome. **c** Genomic collinearity of XX048 haploid genomes with three other species of Euphorbiaceae. **d** Evolutionary scenario of Euphorbiaceae from the AEKs of protochromosomes. The predicted WGD and the WGT-γ events are indicated on the phylogenetic tree. **e** Histogram of the insertion time of LTR-RTs in *M. esculenta* XX048 haplotype genomes, *M. esculenta* AM560-2, *M. esculenta* SC205, *H. brasiliensis*, and *M. annua*.

At present, only five species within the Euphorbiaceae family have annotated reference genomes available, namely *Mercurialis annua*, *Ricinus communis* [18], *Jatropha curcas* [19], *Hevea brasiliensis* [20], and *M. esculenta* [17]. Due to the limited number of reference genomes, the study combined reference genomes from a total of 13 species within the Malpighiales order. A comparative genomic analysis was conducted using rice [23] and *Arabidopsis* [24] as outgroups. The results of the phylogenetic analysis revealed that cassava was positioned lower in the evolutionary hierarchy within Euphorbiaceae. Of the *Euphorbia* species studied, it was found that rubber is the closest relative to cassava, with an estimated divergence time ~34.57 million years ago (Mya), which is consistent with a previous estimation [17] (Fig. 2a). Following this, an analysis was conducted on the synonymous substitution rate (*K*_s_) distribution of homologous gene pairs within and between genomes of various species in the Euphorbiaceae family. The results indicated that the divergence time of cassava and rubber almost coincided with the recent occurrence of whole-genome duplication (WGD) events experienced by species in the Euphorbiaceae family (Fig. 2b). As a result, the calculated divergence times between cassava XX048 and the other three Euphorbiaceae family species, *J. curcas*, *R. communis*, and *M. annua*, were 68.0, 72.2, and 80.6 Mya, respectively (Supplementary Data Fig. S7a). It was demonstrated by gene family analysis of various species in the Euphorbiaceae family that cassava exhibits a higher proportion of rapidly evolving gene families compared with other species within the same family (Fig. 2a). Furthermore, strong synteny was revealed among the species studied in the Euphorbiaceae family, with the number of chromosomes in the species found to progressively decrease with evolutionary status. Specifically, cassava and its closest relative, rubber, had 18 chromosomes, while castor bean had 10 chromosomes, and annual mercury, with a higher evolutionary status, had only 8 chromosomes (Fig. 2c). To further explore this phenomenon, a thorough analysis of the evolutionary history of Euphorbiaceae species has been conducted. First, analysis of WGD events within Euphorbiaceae species was conducted, revealing that all Euphorbiaceae species have undergone a recent WGD event and an ancient whole-genome triplication (WGT-γ) event. The collinearity within the genomes of all species further confirmed the recent WGD event. Notably, the retention of traces of WGT-γ events in the *R. communis* genome was more complete than in other Euphorbiaceae species and could still be clearly observed (Fig. 2b, Supplementary Data Fig. S7b–e). Moreover, in the study of ancestral eudicot karyotype (AEK) composition in Euphorbiaceae species, it was discovered that species with higher chromosome numbers experienced a greater frequency of ancestral chromosome fissions and fusions during karyotype formation. As a result, the constant occurrence of chromosome fissions and fusions throughout the evolutionary process is considered the reason for higher chromosome numbers observed in species with lower evolutionary status in Euphorbiaceae (Fig. 2d). The XX048 genome, consisting of two haploid sets, contains a staggering 595 803 208 bp of LTR-RTs. A statistical analysis of the insertion times of these LTR-RTs revealed that continuous amplification of LTR-RT insertions in the XX048 genome began around 1.5 Mya, with a recent burst of activity. Interestingly, there were more LTR-RT insertions in the haploid A genome compared with the haploid B genome, suggesting the former has more active LTR-RTs [25] (Fig. 2e). The temporal distribution of LTR-RT insertions exhibited a consistent trend across the three cassava varieties (AM560-2, SC205, and the two haploid genomes of XX048 that were assembled). Additionally, the LTR-RT insertions in *H. brasiliensis* and *M. annua* displayed a similar distribution pattern over time. However, it was found that the number of LTR-RT insertions in the *H. brasiliensis* genome was comparable to that in the cassava genome, whereas the *M. annua* genome had significantly fewer insertions than each of the other species analyzed, potentially contributing to its smaller genome size (Fig. 2e). Furthermore, it was observed that LTR-RT insertions exhibited an asymmetric spatial distribution on the two haploid genomes. However, the overall distribution of LTR-RT insertions was concentrated in the vicinity of the centromere region in the middle of the chromosome, with a low number of regions on both sides of the chromosome (Supplementary Data Fig. S8a and b).

### Variation diversity between XX048 haplotypes and cassava varieties

**Figure 3:**
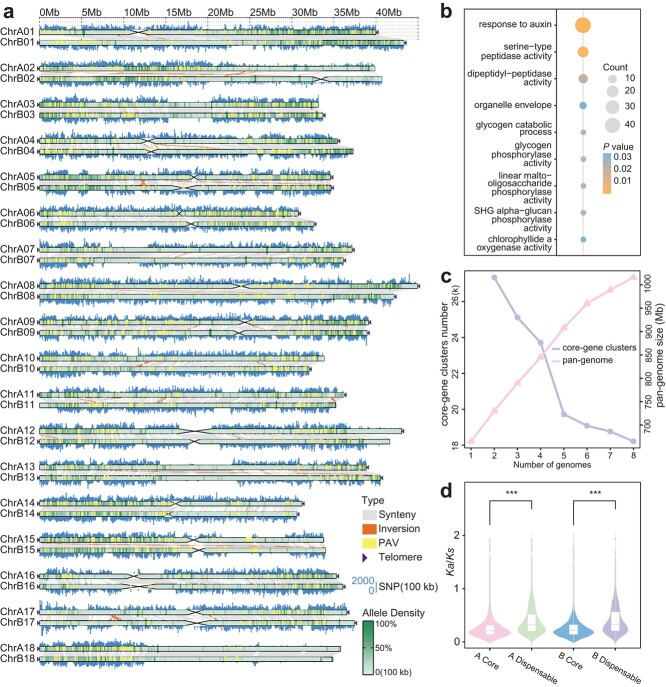
The large number of variations in the cassava genome. **a** The collinear visualization map shows structural variation and other annotated information between homologous chromosomes within the XX048 diploid genome. **b** Highly heterozygous region of GO enrichment in the XX048 genome. **c** Graph-based pan-genome size and the number of core gene clusters. As more genomes were added, the size of the graph-based pan-genome increased and the number of core gene clusters decreased. **d***K*_a_*/K*_s_ within core and dispensable genes. The *t*-test was used to determine significance.

The high level of heterozygosity in cassava XX048 prompted us to carry out a statistical and enrichment analysis of haplotype variation in the cassava genome. A collinear comparison of two haplotype-resolved genomes revealed abundant variation between haplotypes (Supplementary Data Table S7), comprising 3 178 815 single-nucleotide polymorphisms (SNPs), which account for ~0.48% of all bases in the haplotype and 0.62% of all bases in the comparison region. This finding is consistent with the initial estimation of genomic heterozygosity. We observed a higher frequency of alleles in regions of the genome that had a high density of SNPs, as well as a greater presence of alleles at one end of certain chromosomes (Fig. 3a). Furthermore, we annotated the SNPs and tallied the number of genomic functional regions that were affected by them (Supplementary Data Table S8). Minigraph (v0.20) [26] was used to compare the two haplotype-resolved genomes and generate structural variations (SVs) between haplotypes, with a total of 36 956 SVs (variation length >50 bp). Upon analyzing the SV statistics, we detected that 29 847 SVs contained TE sequences, making up 80.76% of the total SVs. This indicates that TE insertion plays a significant role in SV generation, in agreement with previous research. Translocations between chromosomes 1 and 2 were identified by genome-wide collinearity analysis (Supplementary Data Fig. S9a). HiFi reads (Supplementary Data Fig. S9b) and Hi-C reads (Supplementary Data Fig. S9c) were used to verify this translocation. Using haplotype B as a reference, the 10% regions with the highest SNP density in the whole genome were screened and identified as highly heterozygous regions (Supplementary Data Fig. S10a). A total of 4830 genes were identified within the highly heterozygous region. Functional enrichment analysis showed that 44 genes in the highly heterozygous region were associated with the function of auxin response (Fig. 3b), suggesting differences between the two sets of haplotypes in the regulation of auxin response.

To gain a comprehensive insight into the diversity of the cassava genome, we have chosen all cassava genomes assembled from three generations of sequencing technology, and have constructed the pan-genome of cassava through homology-based and graph-based strategies, respectively. As the number of genomes increases, the number of core gene clusters in the cassava pan-genome decreases, while the size of the graph-based pan-genome increases. However, neither of these two indicators appears to have stabilized (Fig. 3c), suggesting that our pan-genome only captures a portion of the diversity between cassava genomes and that there remain unexplored potential diversities. Based on the graph-based pan-genome, we observed that the quantitative distribution of SVs was highest for chromosome 1 and lowest for chromosome 17, with a difference of 1.79 times between them. Moreover, the SV length distribution of each chromosome was largely consistent (Supplementary Data Fig. S10b, Supplementary Data Table S9). Furthermore, the non-synonymous/synonymous substitution ratios (*K*_a_*/K*_s_) of core genes appeared to be significantly lower than those of optional genes (Fig. 3d), suggesting the conservative nature of core genes in evolution. In the functional enrichment of GO, core genes are enriched into a wide range of functions involved in encoding plant growth and development (Supplementary Data Fig. S10c). The analysis of the cassava pan-genome represents the high diversity of the cassava genome, and the development of pan-genome resources will provide a new starting point for the utilization of cassava germplasm resources.

### Differential expression of alleles in haploid genomes

**Figure 4:**
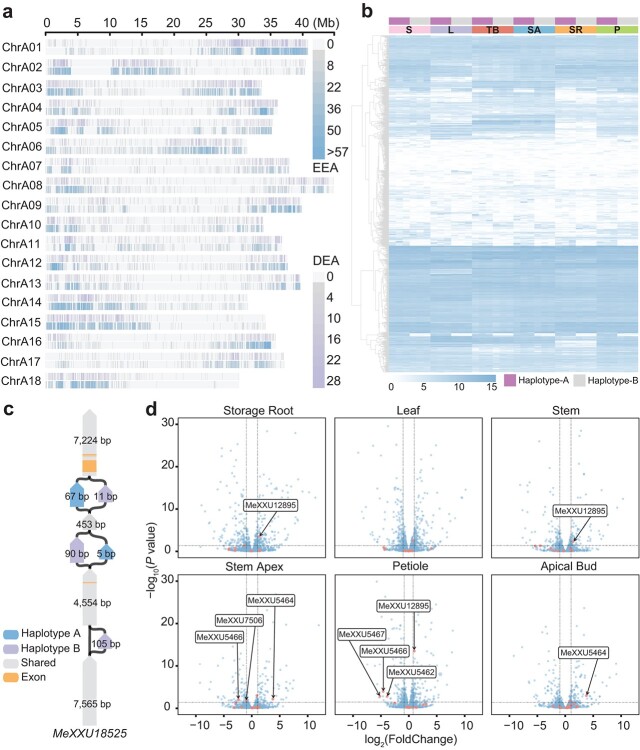
Differential expression of alleles in the cassava XX048 genome. **a** Distribution of DEAs and EEAs in the XX048 genome. **b** Allelic expression profiles of DEAs on chromosome 1 in various tissues (S, stem; L, leaf; TB, terminal bud; SA, stem apex; SR, storage root; P, petiole). DEAs in at least one tissue are shown as a heat map using the log_2_(CPM) value. Heat maps of other chromosomes are shown in Supplementary Data Fig. S10. **c** Graph-based genome visualization of *MeXXU18525*, which encodes glucan endo-1,3-β-glucosidase 4, as well as SVs between the two haploids within and around this gene. **d** Volcano plots depicting highly heterozygous gene expression in various tissues of the XX048 genome. The labels indicate alleles that exhibit differential expression among them. The horizontal dashed line represents log_10_(0.05), while the vertical line represents log_2_(FoldChange) = 2.

Using the two assembled haplotype-resolved genomes, we conducted a comparative analysis of transcriptome data from six tissues of the cassava variety XX048, including root, stem, leaf, terminal bud, stem apex, and petiole. Interestingly, high-density differentially expressed alleles (DEAs) were found in specific regions of both haploid genomes of XX048, such as the ~29 to ~39 Mb region of chromosome 1, in line with observations made in the lychee genome [27]. Additionally, DEA-enriched regions tended to have a higher enrichment of equally expressed alleles (EEAs), although the distribution of both within this region was inconsistent (Fig. 4a). Differential expression of alleles was found to be widespread across all homologous chromosome pairs in various tissues, with multiple patterns of differential expression observed. Moreover, certain alleles displayed tissue-specific expression characteristics, as depicted in Fig. 4b and Supplementary Data Fig. S11. Furthermore, differences in the number of significantly highly expressed alleles between the two haploid genomes were commonly observed on the chromosomes within each tissue. However, the number of DEAs between the haploid genomes was relatively low across the entire chromosome. Most genes were observed to be expressed at similar levels between the two haploid genomes (Supplementary Data Fig. S12). Additionally, statistical analysis of DEAs among tissues indicated that the number of DEAs in storage roots and leaves was much higher than in other tissues, and this was consistent across both haploid genomes (Supplementary Data Fig. S13). Enrichment analysis of DEAs located in storage roots and leaves revealed that in storage roots five DEAs in the A haploid genome were enriched in the starch and sucrose synthesis pathway. In leaves, seven genes in the A haploid genome were enriched in the carbon fixation pathway in photosynthesis, whereas only a few genes in the B haploid genome were enriched in the photosynthesis pathway (Supplementary Data Fig. S13). Consequently, allele-specific high expression within the A haploid genome is believed to be more significant for photosynthesis and starch synthesis in cassava. The analysis of haplotype inter-collinearity revealed that structural variants were prevailing in one of the DEAs situated in the starch and sucrose synthesis pathway. These structural variants were found within the intronic region of the gene, as well as on both sides of the gene, and were assumed to contribute to the differential expression of the alleles (Fig. 4c). Additionally, the differential expression analysis of alleles within the highly heterozygous regions revealed that differential expression was widespread among the highly heterozygous alleles. Notably, a total of six alleles enriched in the auxin response pathway exhibited differential expression between the haploid genomes (Fig. 4d, Supplementary Data Table S10). This phenomenon further implies the existence of differences in the auxin-related regulatory networks between the two haploid genomes.

**Figure 5:**
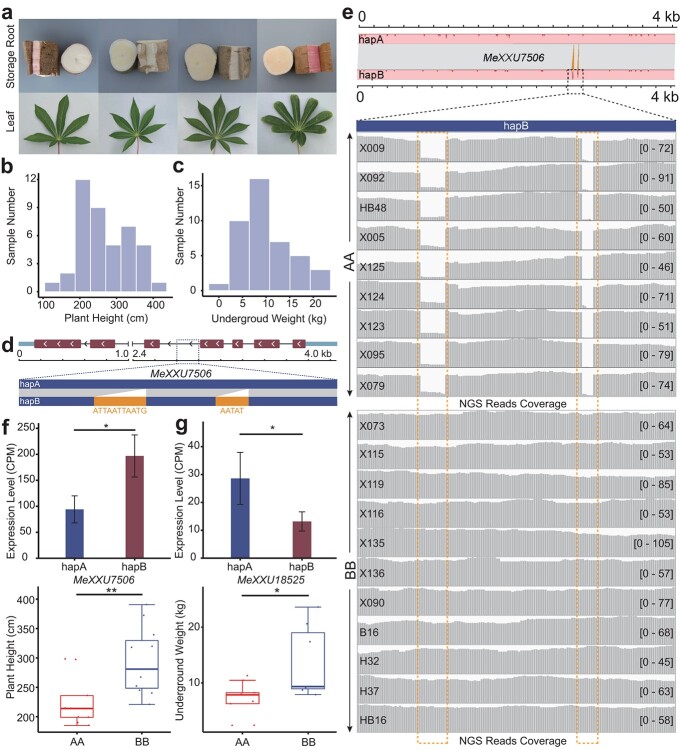
Phenotypic segregation in the *S*_1_ population of XX048. **a** Phenotypic segregation of storage root color, leaflet length, and leaflet count in the XX048 *S*_1_ population. **b** Histogram of distribution of plant height in the XX048 *S*_1_ population. **c** Histogram of distribution of belowground weight in the XX048 *S*_1_ population. **d** Gene structure of *MeXXU7506* showing two InDels between the two haplotypes, with sizes of 11 and 5 bp. **e** Gene typing of *MeXXU7506* in progeny material based on the coverage of Illumina reads within the region of the two InDels between the two haplotypes. **f** Differential expression of *MeXXU7506* between two haploid genomes and significant variations in plant height among inbred lines with different homozygous alleles of this gene. **g** Differential expression of *MeXXU18525* between two haploid genomes and significant variations in underground weight among inbred lines with different homozygous alleles of this gene.

To further investigate the impact of allelic variations in differential gene expression on cassava traits, we conducted a survey of various phenotypic data on 42 lines from the *S*_1_ population of XX048 (Fig. 5a–c, Supplementary Data Table S11). Whole-genome resequencing was performed on these 42 lines (Supplementary Data Table S12), and among them seven lines with significant differences in underground parts were subjected to ONT sequencing, with an average coverage depth of ~34× (Supplementary Data Table S13). In the *S*_1_ population of XX048, several traits were observed to exhibit segregation, such as storage root color, leaflet length, and leaflet count (Fig. 5a). More importantly, trait segregation was observed for plant height and the weight of underground parts in the *S*_1_ population (Fig. 5b and c). In order to investigate the relationship between allelic genotypes and trait differences in *S*_1_ lines, we conducted a strictly genotyping analysis of DEAs in various *S*_1_ lines using Illumina and ONT read mapping methods. Specifically, we focused on the DEA *MeXXU7506* (GO: auxin-activated signaling pathway) in the stem apex, which exhibited two InDel variations between the two haploid genomes (Fig. 5d and e). Notably, there was a significant difference in plant height among lines with different homozygous allele genotypes of *MeXXU7506*. These findings suggest a potential association between this gene and plant height in cassava (Fig. 5e and f, Supplementary Data Fig. S14). Additionally, lines with the BB homozygous genotype containing the gene *MeXXU18525* (KEGG: ko00500), annotated to the starch–sucrose synthesis pathway, exhibited a significantly higher underground weight compared with lines with the AA homozygous genotype. This indicates a potential correlation between this gene and the yield of cassava (Fig. 5g, Supplementary Data Fig. S15). These results indicate that heterozygous genes in the parents contribute to the rapid trait segregation in the *S*_1_ population.

### Differences in epigenetic characterization between haploid genomes

**Figure 6:**
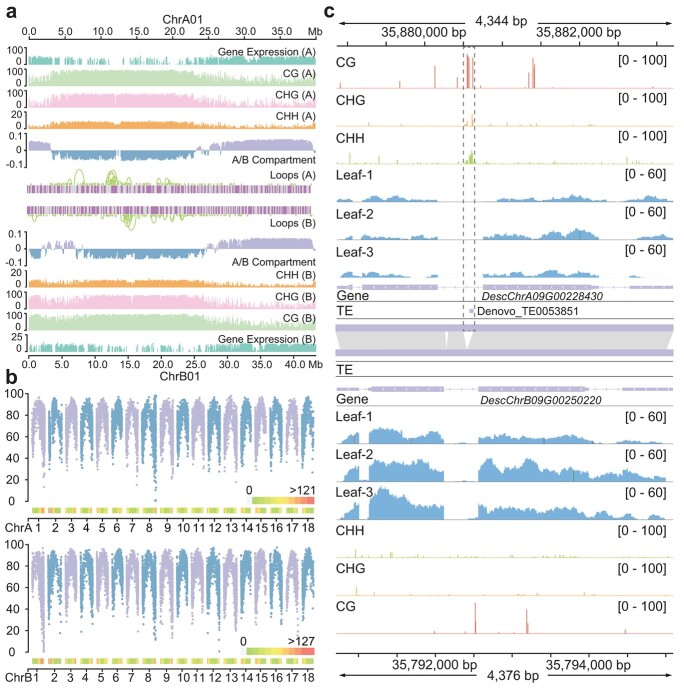
Differences in epigenetic characteristics between haploid genomes of XX048. **a** Distribution of transcriptomic and epigenomic features (DNA methylation and 3D genome) is shown for homologous chromosome 1. **b** Mean CG methylation levels within alleles. The upper section depicts the average CG methylation levels within alleles in the A haploid genome, whereas the bottom section depicts the average CG methylation levels within alleles in the B haploid genome. The heat map depicts the allele density on each chromosome of the haploid genome. **c** Visualization of representative examples of TE insertions that result in SV formation with varied methylation effects on allele expression levels using the Integrative Genomics Viewer (IGV).

To investigate and compare the epigenetic characteristics of the haplotype-resolved XX048 genome, Hi-C and bisulfite sequencing (BS-seq) data [2] from leaves were utilized. This approach allowed a comprehensive epigenetic analysis of both haploid genomes. Both haploid genomes show a pattern of high methylation levels in the repetitive regions around the centromeres, and low levels on both sides, as seen from the distribution of cytosine methylation levels in the three types (CG, CHG, and CHH). Nevertheless, the distribution of methylation levels differs between the two haploid genomes. These regions were consistent in their boundaries and agreed with previous observations of various plant species, including cassava [28, 29]. The regions with high cytosine methylation density were predominantly classified as B compartments, characterized by highly compact chromatin structures. Additionally, the genes located within these regions exhibited lower expression levels than those located in adjacent regions. It was observed that the distribution of TEs was greater in the central centromeric region of the chromosome and lower in the proximal regions of both haploid genomes of XX048. The regions with a high density of TE distribution were consistently found to be spatially aligned with high levels of all three types of cytosine methylation enrichment on all chromosomes (Supplementary Data Fig. S16). In the XX048 genome, a large number of chromatin loops were identified with their two anchor points overlapping with the topologically associated domain (TAD) boundary. Moreover, the distribution of chromatin loops on the chromosome was found to be more enriched in the middle region of the chromosome and less abundant in both arms (Fig. 6a).

Epigenetic variations between the two haploid genomes of XX048 were studied in further detail with a higher resolution. The results revealed distinct differences in several aspects between homologous chromosomes in cassava XX048, including A/B compartment division, TAD division, loop distribution, and cytosine methylation levels. Focusing on homologous chromosome 1, we observed that the region ranging from 2 to 7 Mb on the haploid B genome was classified as A compartment. This region exhibited lower methylation levels and higher transcriptional activity compared with the surrounding regions. However, on the haploid A genome, the corresponding region displayed a different pattern. Only a small portion of it was categorized as A compartment, while the majority of the region fell into B compartment, characterized by higher cytosine methylation levels and lower gene expression levels. Additionally, the distribution of chromatin loops in this region varied from the corresponding region in haploid B genome. The alignment between A/B compartment division and cytosine methylation levels further supported the reliability of the epigenetic data (Fig. 6a). A total of 3679 loops (Supplementary Data Table S14) were identified in the XX048 haploid genome, of which 1899 were located in the haploid A genome and 1780 in the haploid B genome. Notably, the number of loops was also found to be differentially distributed among homologous chromosomes (Supplementary Data Fig. S17a). In the highly heterozygous regions of homologous chromosome 1, a higher resolution analysis revealed significant epigenetic differences between the two haploid genomes in addition to substantial sequence differences. Specifically, haploid B exhibited a higher level of cytosine methylation relative to haploid A, and the two haploid genomes had different distributions of TADs and loops within this region (Supplementary Data Fig. S18). The average cytosine methylation levels within the alleles exhibited a spatial trend of increasing with decreasing distance relative to the centromere region. Notably, differences in the distribution of methylation levels of alleles in the two haploid genomes were observed across multiple pairs of homologous chromosomes, such as in homologous chromosome 1, where more genes with lower methylation levels were found in haploid B (Fig. 6b). The average cytosine methylation levels within the alleles exhibited a spatial trend of increasing with decreasing distance relative to the trophectodomain region (Fig. 6b, Supplementary Data Fig. S17b).

Through statistical analysis of differential methylation between alleles, we found that the number of differentially methylated alleles (DMAs) with higher methylation levels in CG and CHG types differed significantly between the two haploid genomes. The A haploid genome had nearly twice as many DMAs with higher methylation levels as the B haploid genome (Supplementary Data Fig. S17c), suggesting that alleles have a higher methylation level in the A haploid genome. Enrichment analysis of DMAs between the two haploid genomes revealed that alleles with significantly higher levels of methylation in either haploid A or B were enriched in the photosynthetic pathway. In haploid B, DMAs were also found to be enriched in carbon fixation as well as in the MAPK signaling pathway in the plant (Supplementary Data Fig. S17d). Therefore, it is possible that the differential methylation of alleles in cassava leaves is related to photosynthesis in cassava leaves and their resistance to biotic and abiotic stresses. By integrating epigenetic data with allele differential expression data, it was discovered that a substantial proportion (35.60%) of the alleles differentially expressed in leaf tissue had significant methylation differences in the gene body or the upstream and downstream 2 kb regions. Moreover, Fisher’s exact test results indicated that allele differential methylation was significantly associated with the differential expression of alleles (*P* = .02815). Therefore, differential methylation of alleles was considered to be one of the factors contributing to the generation of differential allele expression. Statistical analysis of TE insertions in gene bodies with alleles differing in CG-type methylation levels revealed that TE insertions were present in the majority (81.87%) of gene bodies with alleles differing in CG-type methylation. Additionally, differences in TE insertions between some of these alleles were observed to coincide with changes in methylation levels occurring between alleles. Notably, the differences in TE insertion among some of these alleles were also spatially consistent with changes in methylation levels among the alleles, accompanied by differential expression of the alleles (Fig. 6c, Supplementary Data Fig. S17e). The Pearson correlation test indicated that the difference in insertion length of TEs between two alleles in the gene body was positively correlated with the difference in CG methylation rate between the alleles in the gene body (*P* = 2.2e−16) (Supplementary Data Fig. S17f). Therefore, the difference in TE insertion in the gene body between alleles is considered to be one of the factors contributing to the generation of methylation differences among cassava alleles.

## Discussion

Based on the near-complete assembly of two sets of haplotype-resolved genomes from XX048, we have, for the first time, conducted an analysis of karyotype evolution in Euphorbiaceae species, including cassava. Significant differences in chromosome numbers were observed among the Euphorbiaceae species examined, with those possessing a lower evolutionary status exhibiting a greater number of chromosomes. This was attributed to differences in the frequency of chromosome fusion and fission between the Euphorbiaceae species, with species of a lower evolutionary status experiencing a higher number of fusions and fissions, ultimately resulting in a greater number of chromosomes. Through the investigation of differences between the XX048 haploid genomes, we observed significant discrepancies in the allelic genome in response to auxin, starch synthesis, and photosynthesis. Our examination of these crucial pathways and genes provides a basis for the identification of candidate genes in the subsequent haploid molecular breeding of cassava.

Combining epigenetic analysis with TE insertion, it was determined that the main source of structural variation between haploid genomes was TE insertion. Specifically, the differences in TE insertion between the gene bodies of alleles were thought to contribute to the differences in CG methylation levels between alleles. Furthermore, these differences in CG methylation levels between alleles were assumed to be one of the causes of allelic differential expression. Based on these findings, it was speculated that the differential expression of some alleles could be attributed to methylation differences that occurred as a result of different TE insertion.

Currently, breeding has entered the pan-genome era. The pan-genome can characterize most of the variation of a species and be used to analyze the variation differences across the entire population [30]. By associating structural variation with traits, candidate genes related to the phenotype can be quickly identified, thus significantly accelerating the molecular breeding process [31]. Pan-genomic analysis requires a substantial number of high-quality reference genomes [31,32]. Compared with major food crops, such as rice [33] and maize [34], or cash crops, such as radish [35] and rape [25], cassava, which has the third highest edible population in the world, has limited genomic resources. To expedite the molecular breeding of cassava, it is essential to assemble more high-quality cassava genomes as soon as possible. Currently, the main factor limiting the assembly of cassava genomes of high quality is the large size of the genome and its high level of heterozygosity. With the improvement of assembly algorithms and the increasing use of new sequencing techniques, such as ONT ultra-long reads, it is anticipated that high-quality cassava genomes will be more easily assembled, enabling more comprehensive analyses of haploid differences.

## Materials and methods

### Plant materials

‘Xinxuan 048’, is a new cassava variety developed by Guangxi University from the natural variation population of germplasm resource ZM93, and then through systematic selection and directional selection. It has strong growth, high yield, good resistance and other good characteristics. The individual ‘Xinxuan 048’ whose genome was sequenced was obtained from the Guangxi Academy of Agricultural Sciences.

### Genome assembly

To estimate the genome size and heterozygosity size of cassava, we utilized *k*-mer analysis on PacBio HiFi reads. Specifically, we employed the Jellyfish (v2.2.10) [36] and GenomeScope2 (v2.0) [37] processes with different *k*-mer length settings. Additionally, we predicted the size of the cassava genome and heterozygosity based on second-generation Illumina reads utilizing the preqc program in sga (v0.10.15) [38]. Next, we use the pipeline method in Supplementary Data Fig. 1d for genome assembly. We combined the PacBio HiFi reads with double-ended Hi-C reads using the hifiasm (v0.16.1-r375) [39, 40]. We utilized the pseudohaploid (https://github.com/schatzlab/pseudohaploid) tool to perform de-redundancy. We used Juicer [41], 3D-DNA (v201008) [41], and JuiceBox (v2.20.00) [42] to complete the assembly of scaffolds. We used LR_Gapcloser [43] and Racon (v1.4.3) [44] with PacBio HiFi reads to fill gaps and correct haplotype-resolved genomes. We used RagTag (v2.1.0) [45] to repair the telomere sequences. Subsequently, we used TGS-GapCloser (v1.2.1) [46] with XX048 progeny ONT reads for gap filling and plion (v1.24) [47] with Illumina paired-end reads for correction. Merqury (v1.3) [48] was used to evaluate the base accuracy of diploid XX048 after gap repair, and homologous chromosomes with higher QV were selected to obtain the XX048 integrated gap-free genome.

### Detection of structural variations

We compared two haplotype genomes using the nucmer program in MUMmer (v4.0.0rc1) [49]. The results were filtered using delta-filter comparison, and variation statistics were analyzed using SyRI (v1.6.3) [50]. Additionally, SnpEff (v5.1d) [51] was employed to annotate the position of SNPs. CMplot (v4.3.0) [52] was employed for visualizing highly heterozygous regions. The haplotype comparison results were visualized using GenomeSyn [53]. Furthermore, we used minigraph (v0.20) [26] to compare two haplotype genomes and extracted the bubble in the gfa file as SVs via gfatools (v0.5) (https://github.com/lh3/gfatools). We checked the authenticity of the chromosome 1 and 2 translocations by comparing the PacBio HiFi reads with the XX048 genome using minimap (v2–2.26-r1175) [54], performed the alignment file processing using SAMtools (v1.15) [55,56], and visualized it using the Integrative Genomics Viewer (IGV v2.16.2) [57]. We also demonstrated the translocation region Hi-C interaction matrix using HiCExplorer (v3.7.2) [58].

### Graph-based pan-genome

We utilized minigraph (v0.20) [26] to construct a graphical pan-genome of cassava by incorporating all current cassava genomes assembled based on triple sequencing technology, with the haplotype A genome serving as a reference. The variation information was analyzed using gfatools (v0.5) (https://github.com/lh3/gfatools). We conducted a statistical analysis of the number and length distribution of SVs <1 kb among cassava varieties. Additionally, the homology annotation of cassava genomes other than XX048 was carried out using gmap (v2021-08-25) [59]. Finally, the construction of the pan-genome gene cluster for the cassava homology strategy was completed via OrthoFinder (v2.5.4) [60, 61].

### Phylogenetic and comparative genomics analysis

We utilized OrthoFinder (v2.5.4) [60, 61] to extract single-copy homologous genes from selected *Euphorbia* species, and RAxML (v8.2.12) [62] was employed to construct phylogenetic trees. Furthermore, CAFE (v4.2.1) [63] was used to analyze gene family expansion and contraction, whereas mcmctree in PAML (v4.9) [64] was utilized for species analysis. Phylogenetic tree mapping was carried out using iTOL (https://itol.embl.de/), and the YN00 model in PAML (v4.9) [64] was employed for calculating *K*_a_ and *K*_s_ values. Covariance analysis of Euphorbiaceae species was performed using MCscan (Python version) [65], and karyotype evolution analysis of Euphorbiaceae species was carried out using WGDI [66]. Additionally, the *Tetracentron sinense* genome [67] was utilized for AEK inference based on the WGDI example file (https://github.com/SunPengChuan/wgdi-example).

### Differential expression of alleles

We conducted differential expression analysis of alleles utilizing transcriptomic data from leaves, petioles, stems, stem apexes, storage roots, and terminal buds. HISAT2 (v2.2.1) [68] was employed for transcriptome sequencing data alignment, while SAMtools (v1.15) [55, 56] was used for processing the alignment results. After processing, featureCounts (v2.0.3) [69] was utilized for transcript quantification. Allelic expression analysis was carried out using DESeq2 [70], and clusterProfiler [71, 72] was used for enrichment analysis of the DAEs. Jvenn [73] was employed for Venn diagramming and allelic SVs were presented using Bandage (v0.9.0) [74].

### Epigenetic differences of alleles

We analyzed BS-seq data based on the methods used in published analyses [29]. The BSMAP [75] alignment to haploid genomes was employed, and MethylDackel (https://github.com/dpryan79/MethylDackel) was utilized for statistical and computational determination of methylation levels from the alignment results. In-house R scripts were used for the statistical analysis of allele methylation levels, and bilateral Fisher tests were employed for the analysis of allele methylation levels. CG and CHG methylated alleles with mean methylation differences of 20% and *P* value <.05 based on the overall methylation levels of CG, CHG, and CHH were defined as differentially methylated genes, whereas CHH methylated alleles with mean methylation differences >5% and *P* value <.05 were also defined as differentially methylated genes. HiC-Pro (v3.1.0) [76] was utilized for Hi-C data analysis and matrix construction, and the resulting matrix was used for 3D genomics analysis and presentation of results via HiCExplorer (v3.7.2) [58]. Additionally, CMplot (v4.3.0) [52] was employed for visualizing allele methylation levels.

### Construction of *S*_1_ population

An *S*_1_ population from the elite cultivar XX048 was constructed after establishing a series of techniques, including flower induction, pollen preservation, breaking seed dormancy, and seedling cultivation. The phenotypic precise identification and evaluation of the *S*_1_ population was based on the description standard for germplasm resources of cassava (NY/T 1943–2010).

## Acknowledgements

This work was supported by the National Natural Science Foundation of China (32100526, 32270712), the Guangxi Natural Science Foundation (AD23026047), the Young Elite Scientists Sponsorship Program by CAST (2022QNRC001), the State Key Laboratory for Conservation and Utilization of Subtropical Agro-Bioresources (SKLCUSA-a202205, SKLCUSA-a03), Ba-Gui Scholar Program of Guangxi (To Z.G. H), the Project of Bama County for Talents in Science and Technology (20220008), Chief Expert of Tuberous Crops Innovation Team in Guangxi Province (nycytxgxcxtd-2023-11-01) and the starting research grant for High-level Talents and Innovation and development multiplication plan from Guangxi University (2022BZRC015).

## Author contributions

J.M.S., L.L.C., and H.B.Y. conceived and designed the study. L.L., X.H.S., P.S., W.Z., S.C., and Z.D.W. contributed to sample preparation. X.D.X., R.P.Z., L.X., M.G., Y.H.L., Z.W.Z., S.Y.Y., Y.Q.Q., and B.L.F. participated in data analysis and substantively revised the manuscript. All authors read and approved the final manuscript.

## Data availability

The original sequencing and assembly of the cassava variety XX048 genome is available from the National Genomics Data Center [77] under project number PRJCA016162.

## Conflict of interest

The authors declare that they have no conflict of interest.

## Supplementary Material

SupplementrayFigure1_18_uhad200

TableS1-14_uhad200

Supplementary_Methods_uhad200
