# Supplementary material for: Telomere-to-telomere assembly of cassava genome reveals the evolution of cassava and divergence of allelic expression
Source: Hortic Res. 2023 Oct 5;10(11):uhad200. doi: 10.1093/hr/uhad200 (PMC10673656; doi:10.1093/hr/uhad200)
Supplement: SupplementrayFigure1_18_uhad200 [file supplementrayfigure1_18_uhad200.zip › SupplementrayFigure1_18_uhad200.pdf]

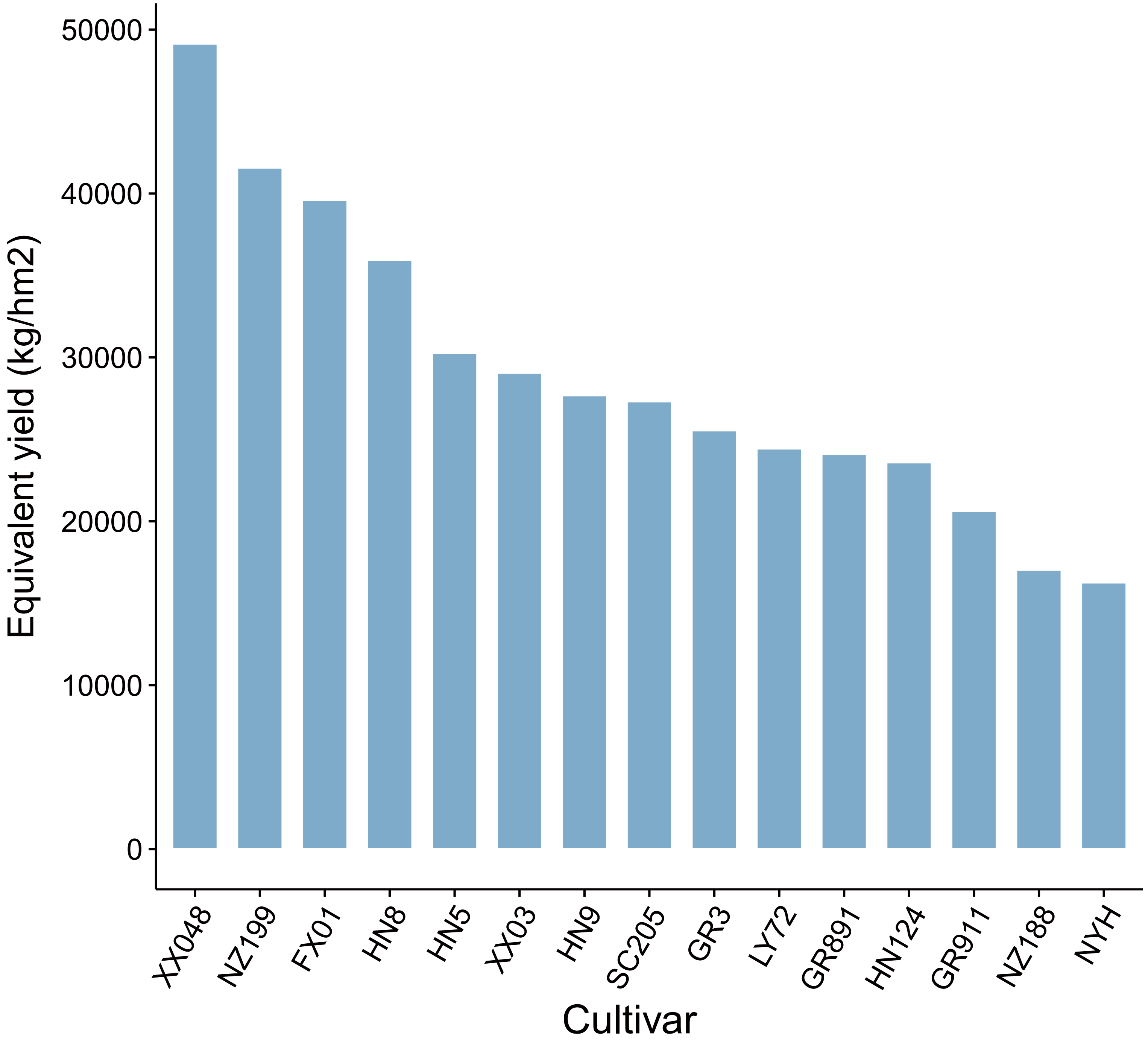

Fig. S1. Comparison of equivalent yield of XX048 with several existing cassava cultivars.

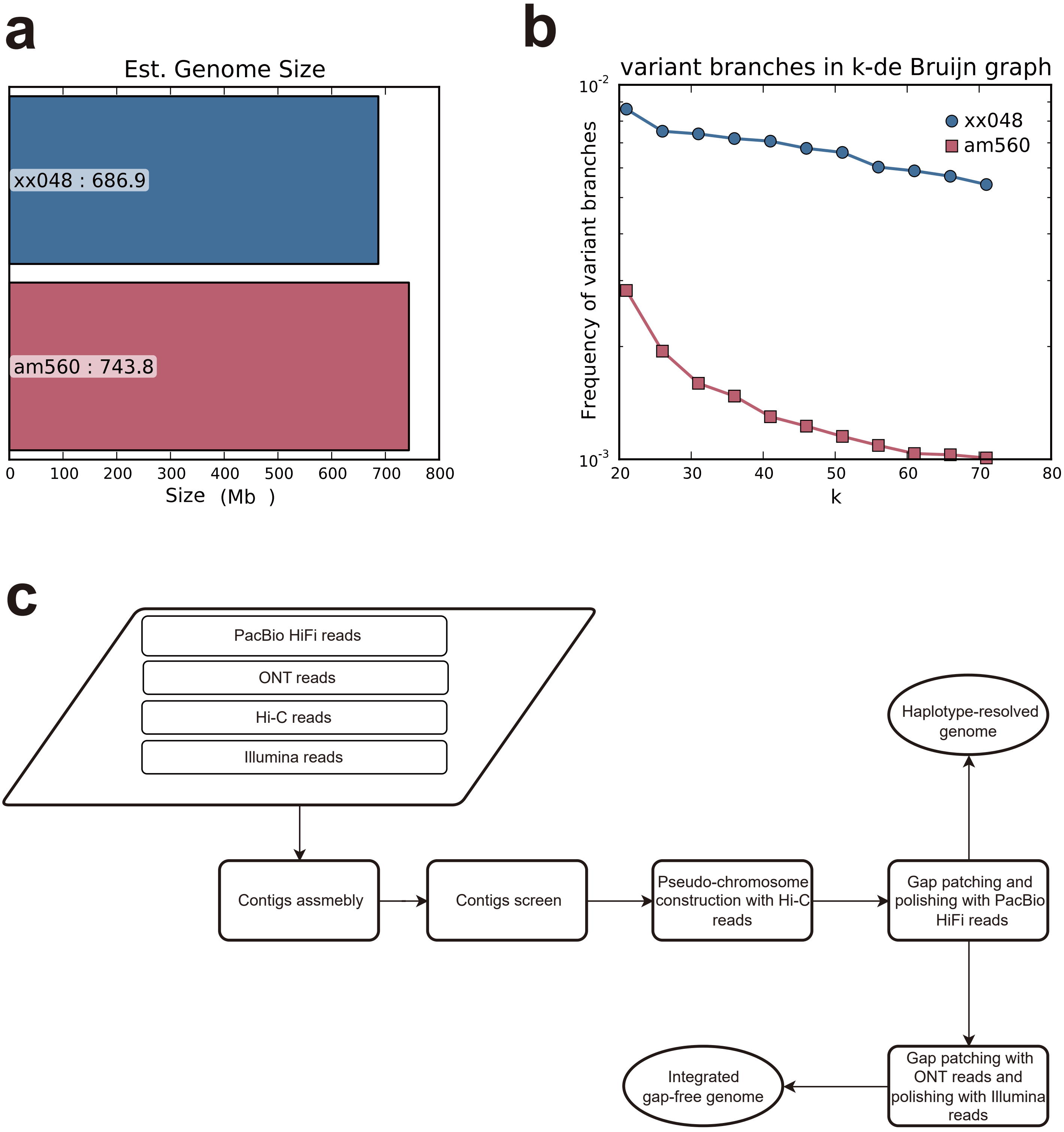

Fig. S2. Genome survey and assembly workflow. (a) Illumina short-read data was used to estimate the genome size of XX048, and am560 was the control. (b) Illumina short-read data was used to estimate the heterozygosity of XX048, and am560 as the control. (c) Overview of the data processing pipeline used for the assembly of XX048 genome.

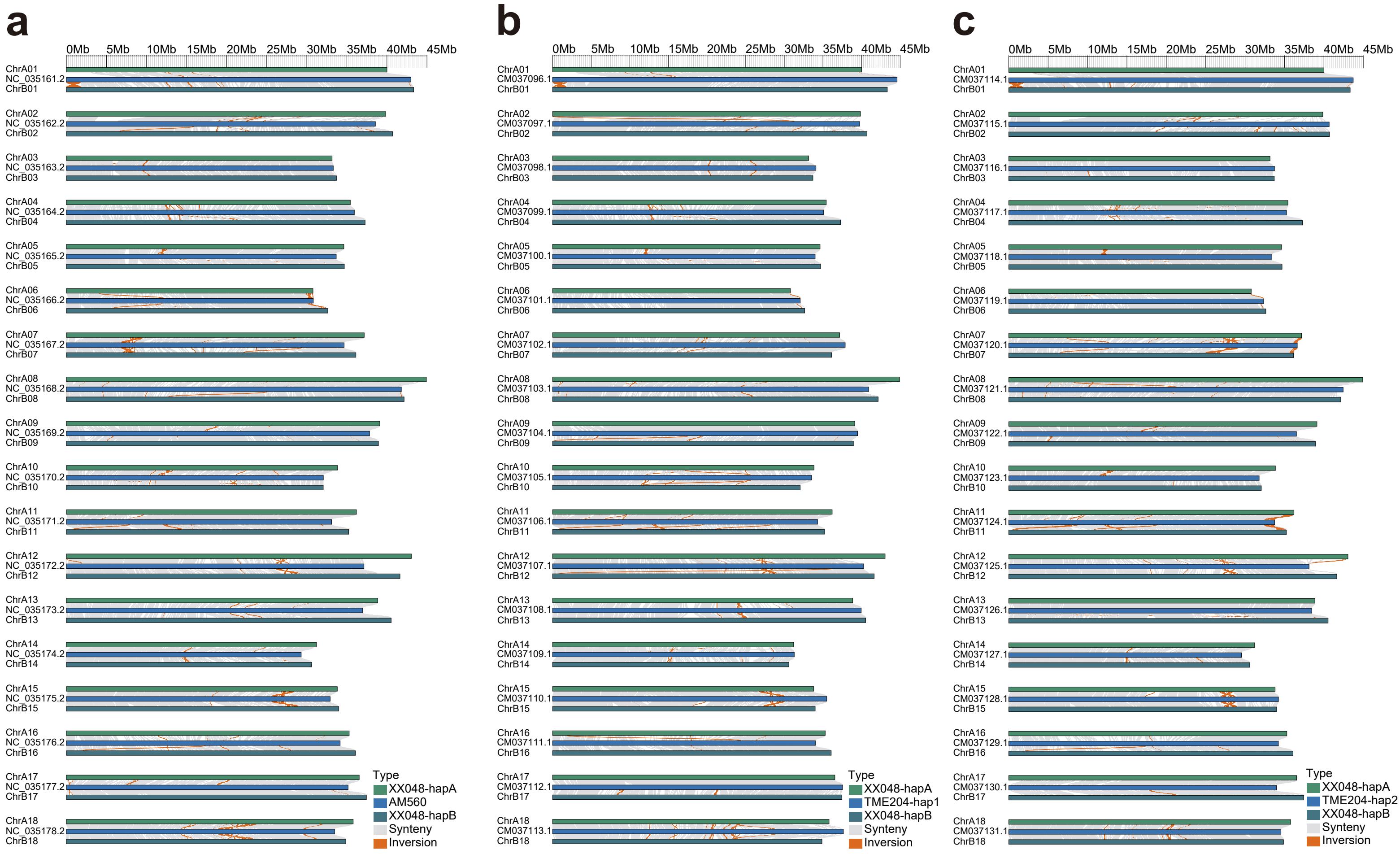

Fig. S3. The collinearity between the cassava XX048 genome and the published cassava genome. a, Collinearity between the XX048 and AM560 genomes. b, Collinearity between the XX048 and TME204-hap1 genomes. c, Collinearity between the XX048 and TME204-hap2 genomes.

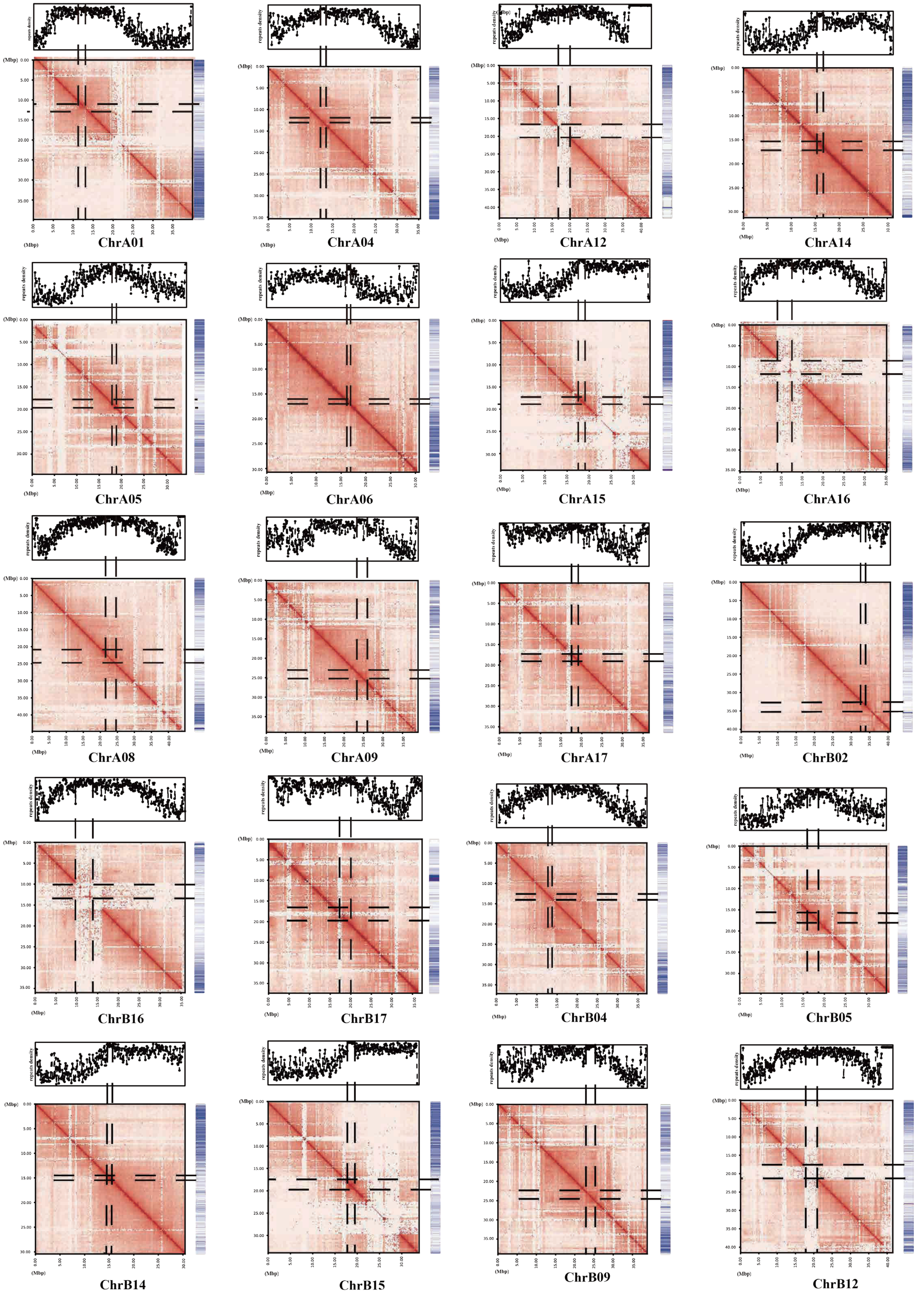

Fig. S4. Candidate centromere region of XX048 whole-genome. Illustrated based on chromatin interaction map, repeat densities, and gene densities.

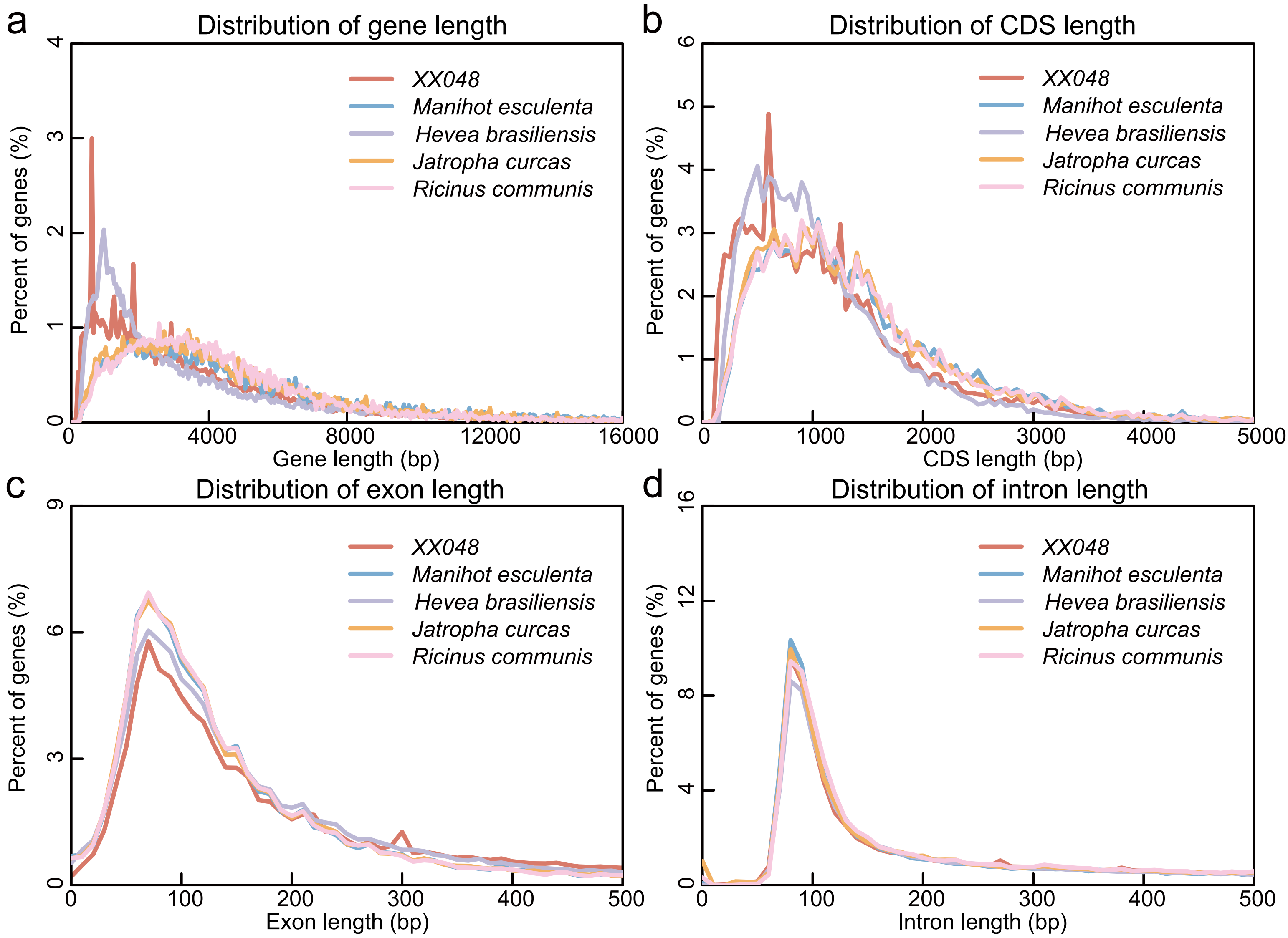

Fig. S5. Distribution of gene elements length. (a) Distribution of gene length. (b) Distribution of CDS length. (c) Distribution of exon length. (d) Distribution of intron length.

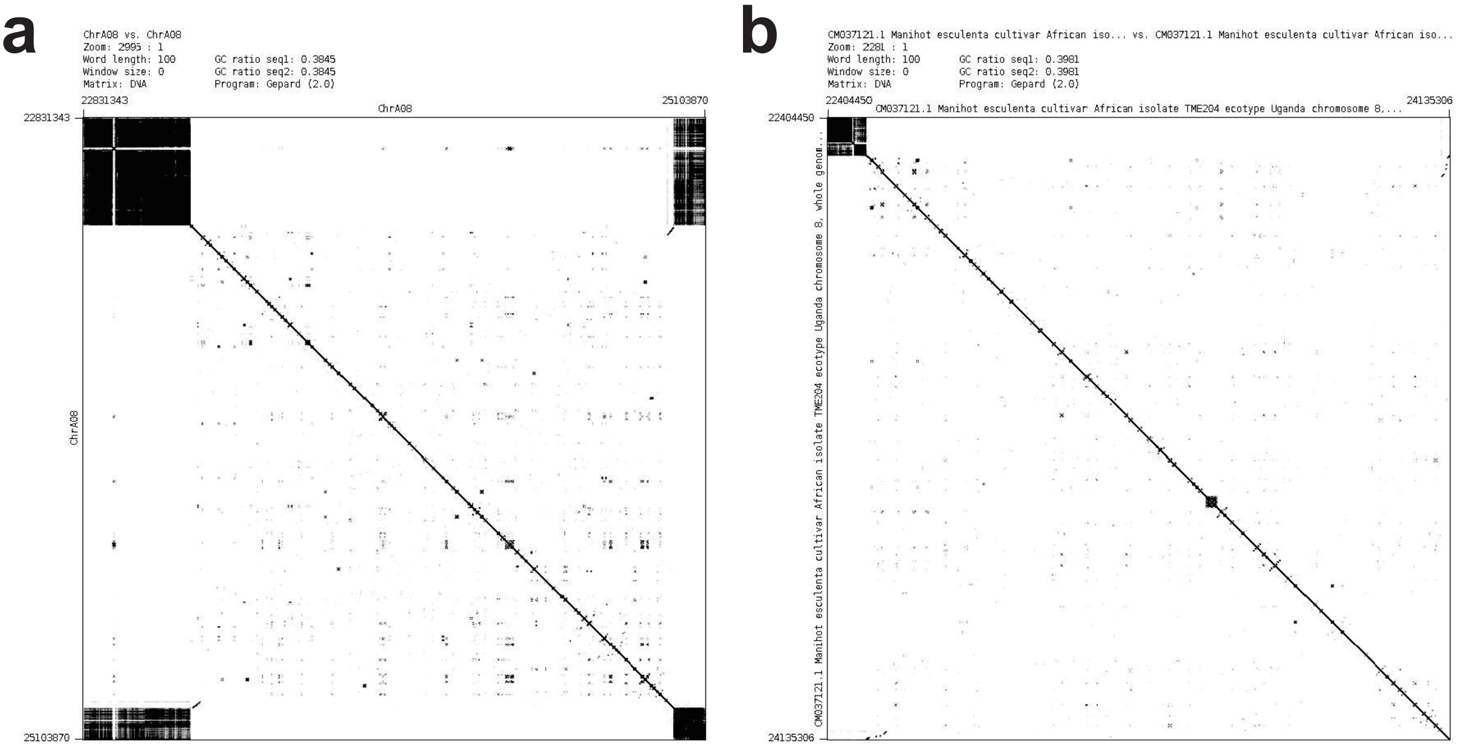

Fig. S6. Dotplot illustrating the distribution of tandem repeats in the centromeric region of chromosome 8 in XX048 hapA and TME204 hap2. (a) XX048 hapA chromosome 8. (b) TME204 hap2 chromosome 8.

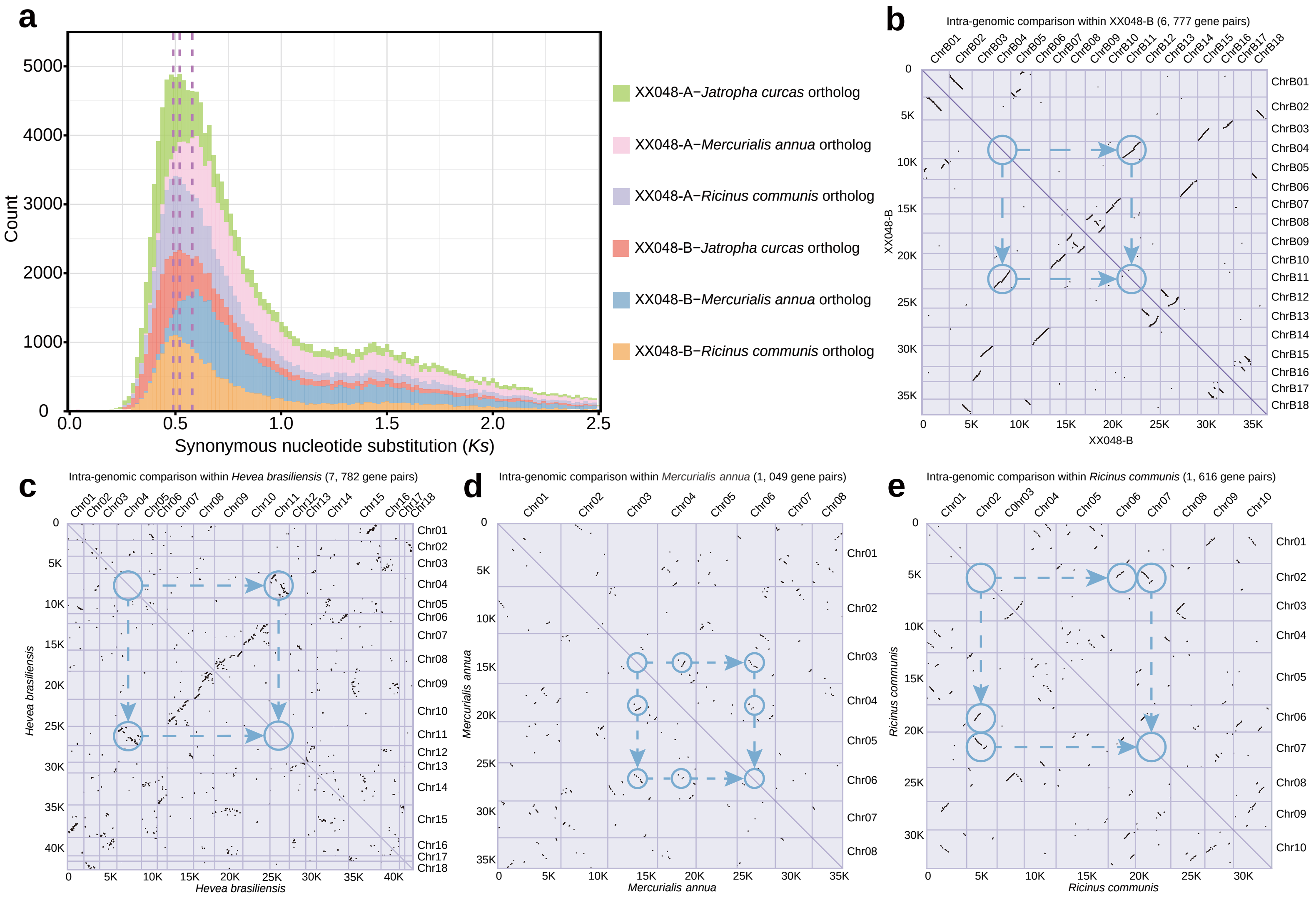

Fig. S7. Whole-genome duplication events experienced by species in the Euphorbiaceae family. (a) *Ks* distribution of XX048 haplotype A− *J. curcas* ortholog (green), XX048 haplotype A−*M. annua* ortholog (pink), XX048 haplotype A−*R. communis* ortholog (purple), XX048 haplotype B−*J. curcas* ortholog (red), XX048 haplotype B−*M. annua* ortholog (blue) and XX048 haplotype B−*R. communis* ortholog (orange). The purple vertical dashed lines mark the locations of the peak *Ks* distribution, from left to right, 0.49, 0.52, 0.58. (b) Dot plots of paralogues in XX048 haplotype B illustrating WGD (1–2 chromosomal relationships in blue circles) events. (c) Dot plots of paralogues in *H. brasiliensis* illustrating WGD (1–2 chromosomal relationships in blue circles) events. (d) Dot plots of paralogues in *M. annua* illustrating WGD and WGT-γ (1–3 chromosomal relationships in blue circles) events. (e) Dot plots of paralogues in *R. communis* illustrating WGD and WGT-γ (1–3 chromosomal relationships in blue circles) events.

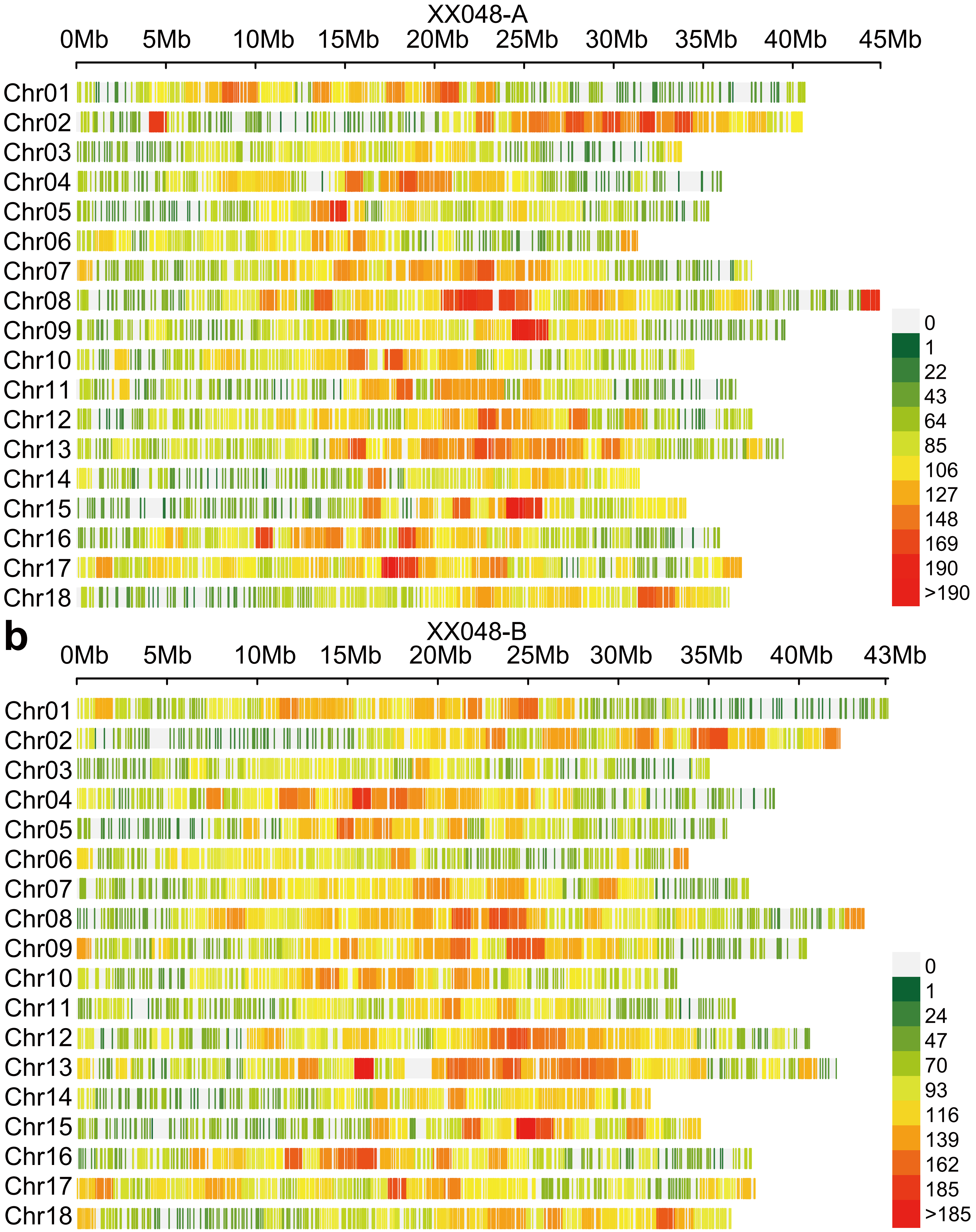

Fig. S8. Spatial distribution of LTR-RT insertions in the XX048 haploid genome. (a) Distribution of LTR-RT insertions on each chromosome of the XX048 haploid A genome. The insertion density of LTR-RT is indicated by a green-to-yellow-to-red color scheme, with warmer colors indicating a higher insertion density of LTR-RT. (b) Distribution of LTR-RT insertions on each chromosome of the XX048 haploid B genome.

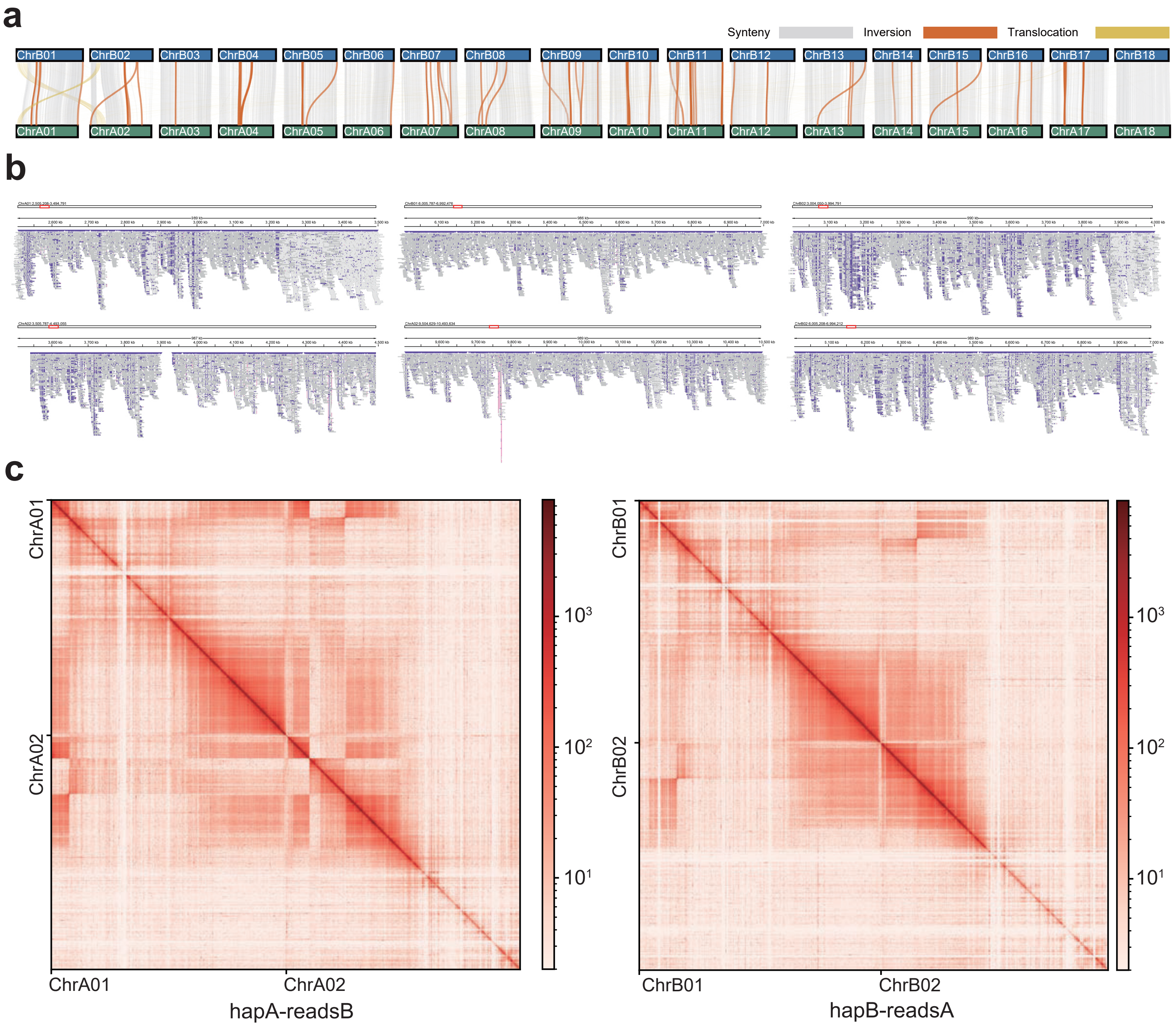

Fig. S9. Discovery and validation of translocations on chromosomes 1 and 2 of XX048. (a) Collinearity of the whole genome. (b) Verification of translocation events between chromosomes 1 and 2 was conducted using PacBio HiFi reads, which were aligned to the whole genome. And confirm that there is continuity of coverage at the commencement and conclusion of translocation. (c) The Hi-C analysis validated a translocation event between chromosomes 1 and 2. Hi-C contact maps at 150-kb resolution for accession XX048 hapA and XX048 hapB, using Hi-C data from the XX048 hapB and XX048 hapA.

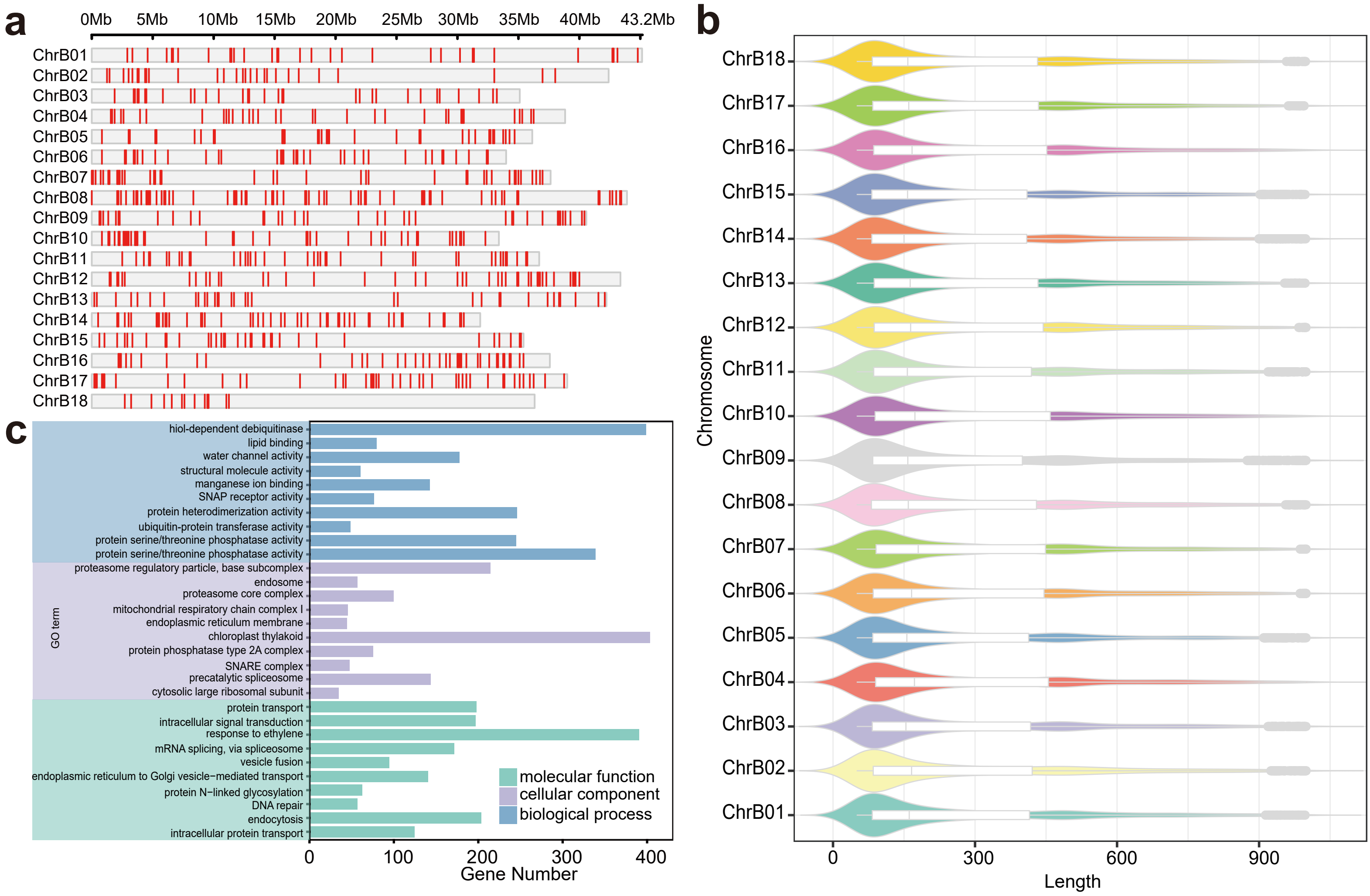

Fig. S10. Highly heterozygous regions and pan-genome statistics. (a) Highly heterozygous regions in the XX048 genome are displayed using XX048 hapB as a reference. (b) The distribution of lengths of structural variants in the cassava graph-based pan-genome (with SV lengths greater than 50 bp, but less than 1 kb). (c) Performing functional enrichment analysis of core genes in the cassava genome.

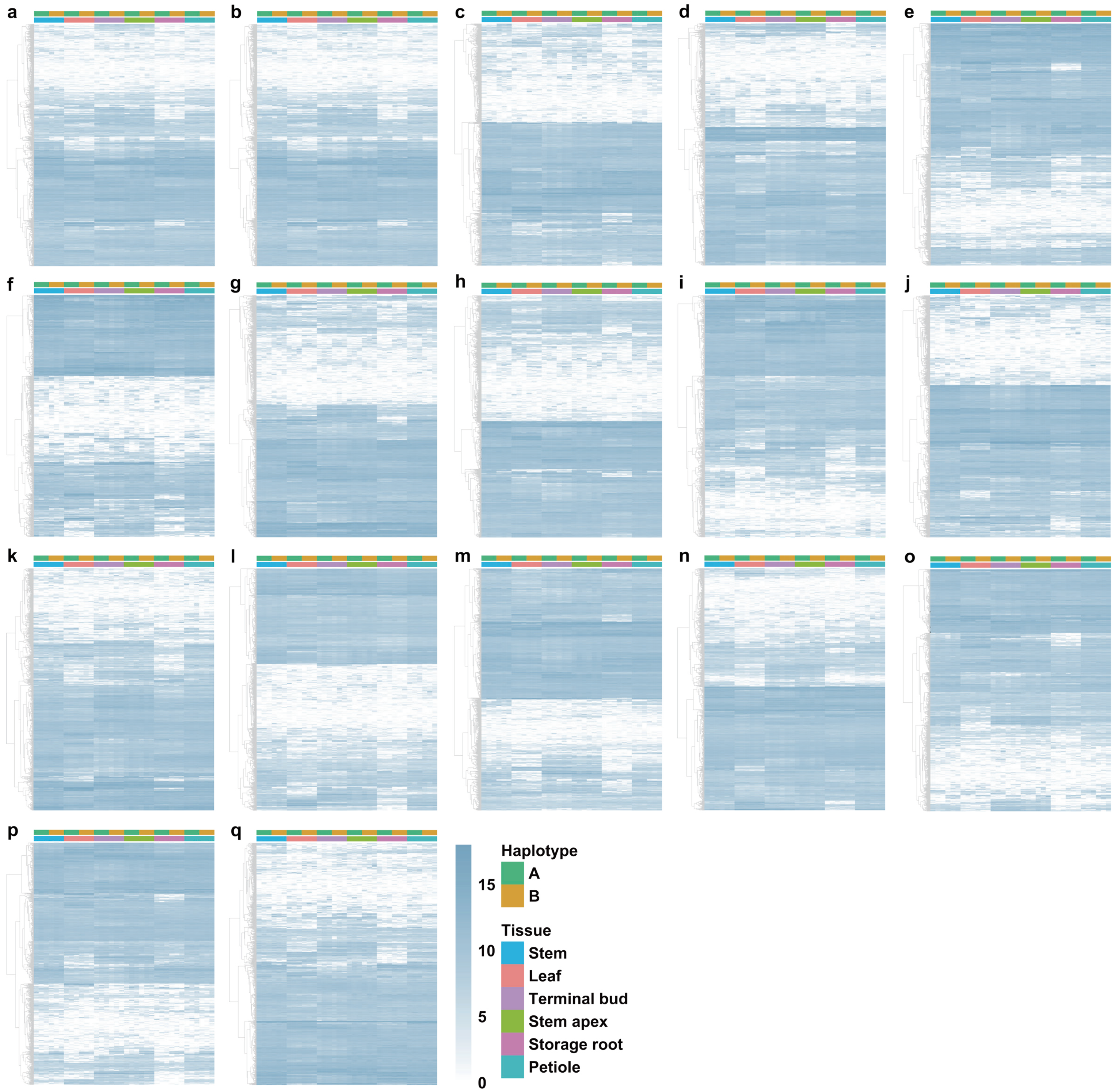

Fig. S11. Differential allele expression profiles of homologous chromosomes 2-18 in various XX048 tissues. Sequential representation of homologous chromosomes 2 to 18, displayed from a to q.

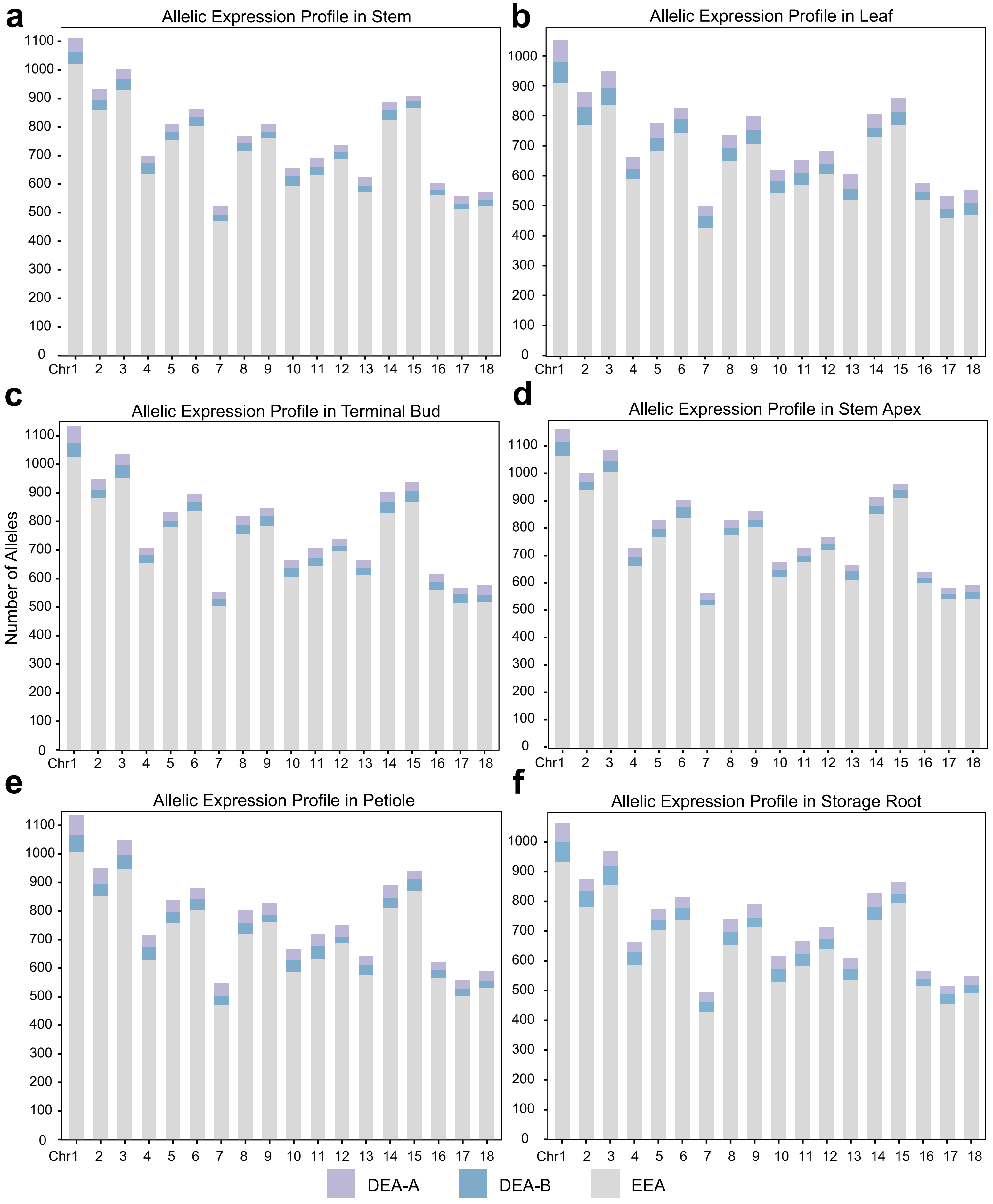

Fig. S12. Composition of DEA and EEA counts on each chromosome across different tissues. The composition of DEA and EEA counts in stem, leaf, terminal bud, stem apex, petiole and storage root, displayed from a to f, respectively.

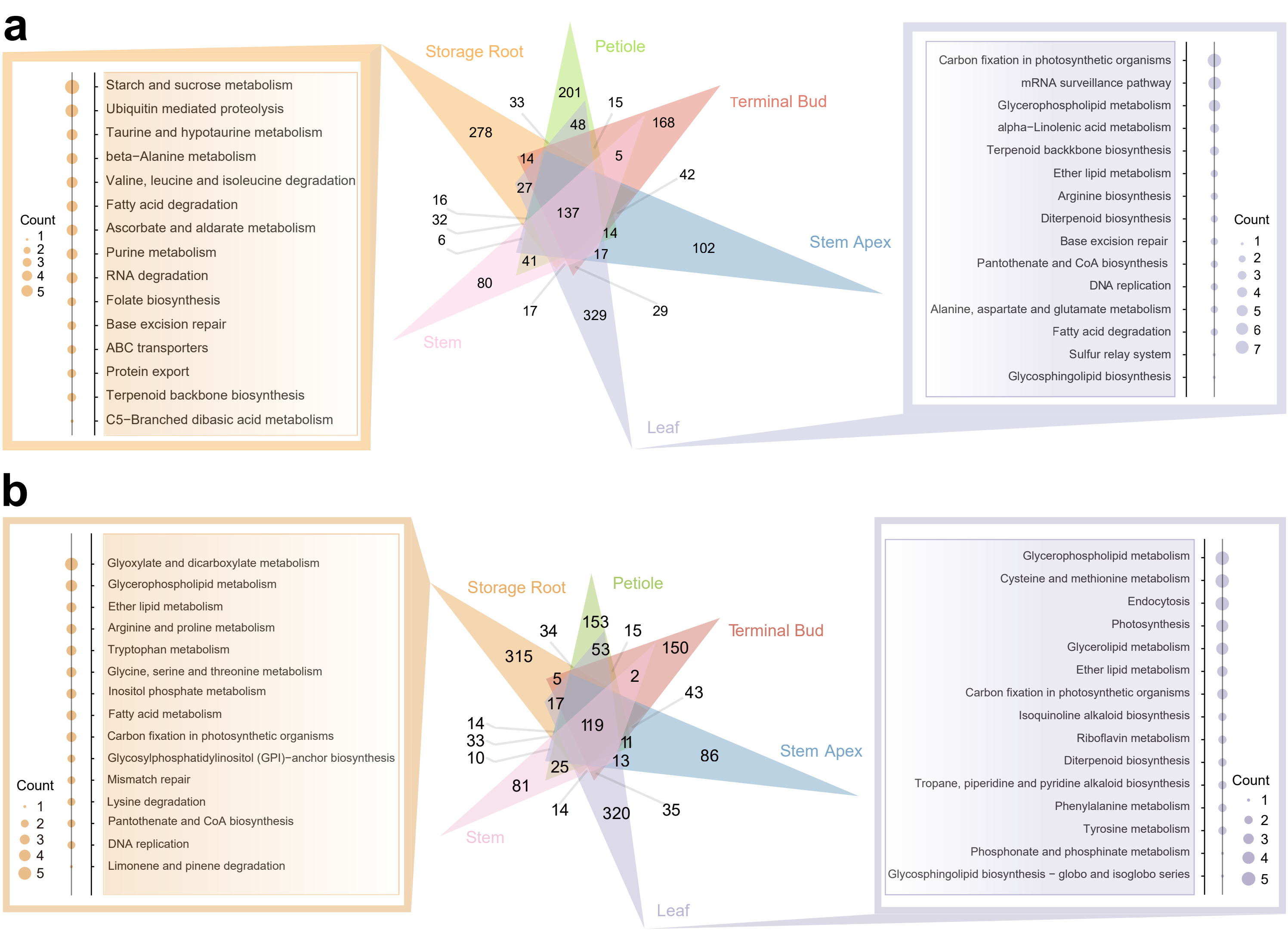

Fig. S13. Comparison of differentially expressed alleles (DEA) in various **tissues, as well as KEGG enrichment of DEA in leaves and storage roots.** (a) Comparison of differentially expressed alleles exhibiting higher expression in A haploid genome (DEA-A) in various tissues, as well as KEGG enrichment of DEA-A in leaf and storage root. (b) Comparison of differentially expressed alleles exhibiting higher expression in B haploid genome (DEA-B) in various tissues, as well as KEGG enrichment of DEA-B in leaf and storage root.

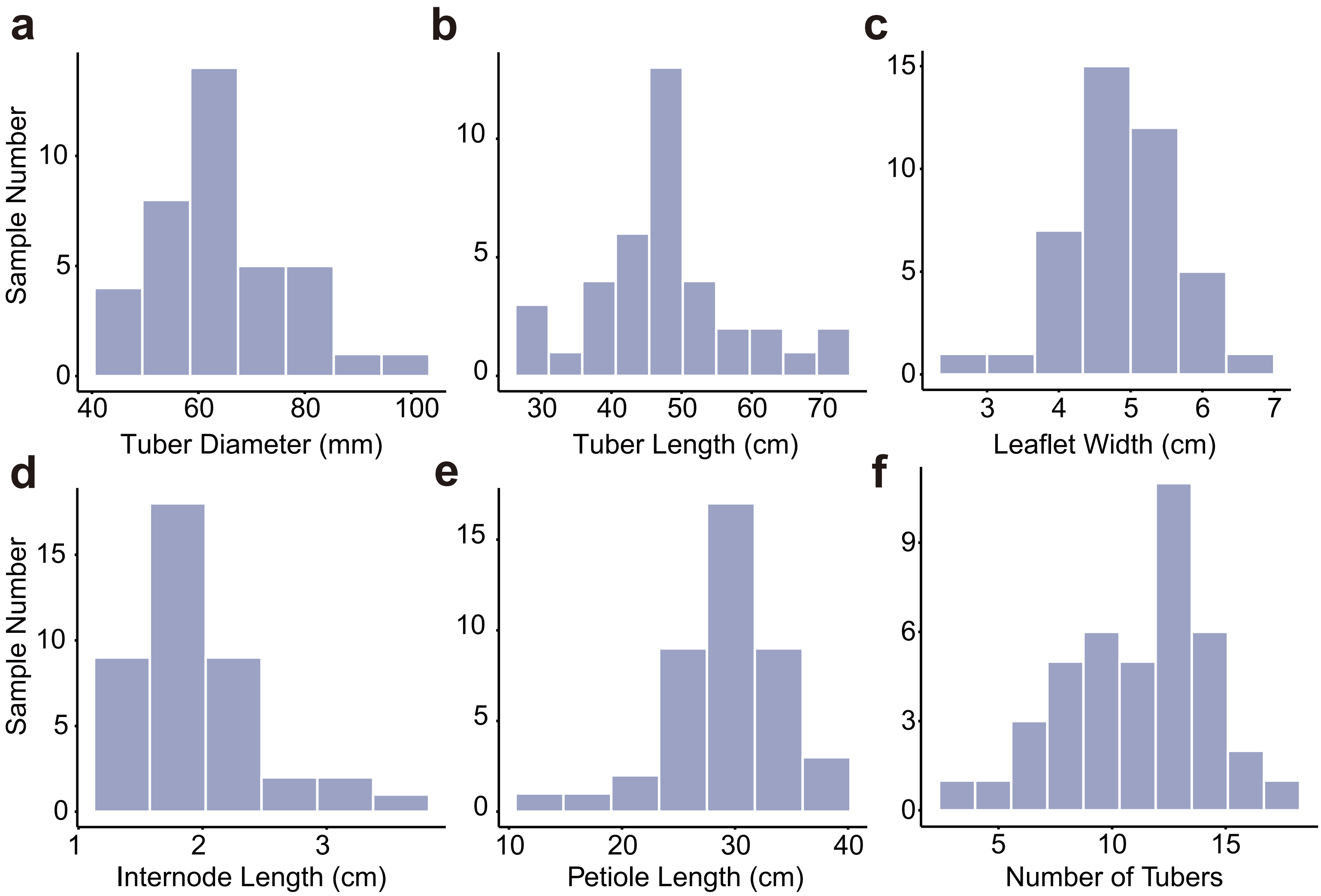

Fig. S14. The segregation of six quantitative traits in the cassava XX048 S_1 population. The graphs illustrate the distribution of the following traits in the S_1 population: (a) tuber diameter, (b) tuber length, (c) leaflet width, (d) internode length, (e) petiole length, and (f) number of tubers.

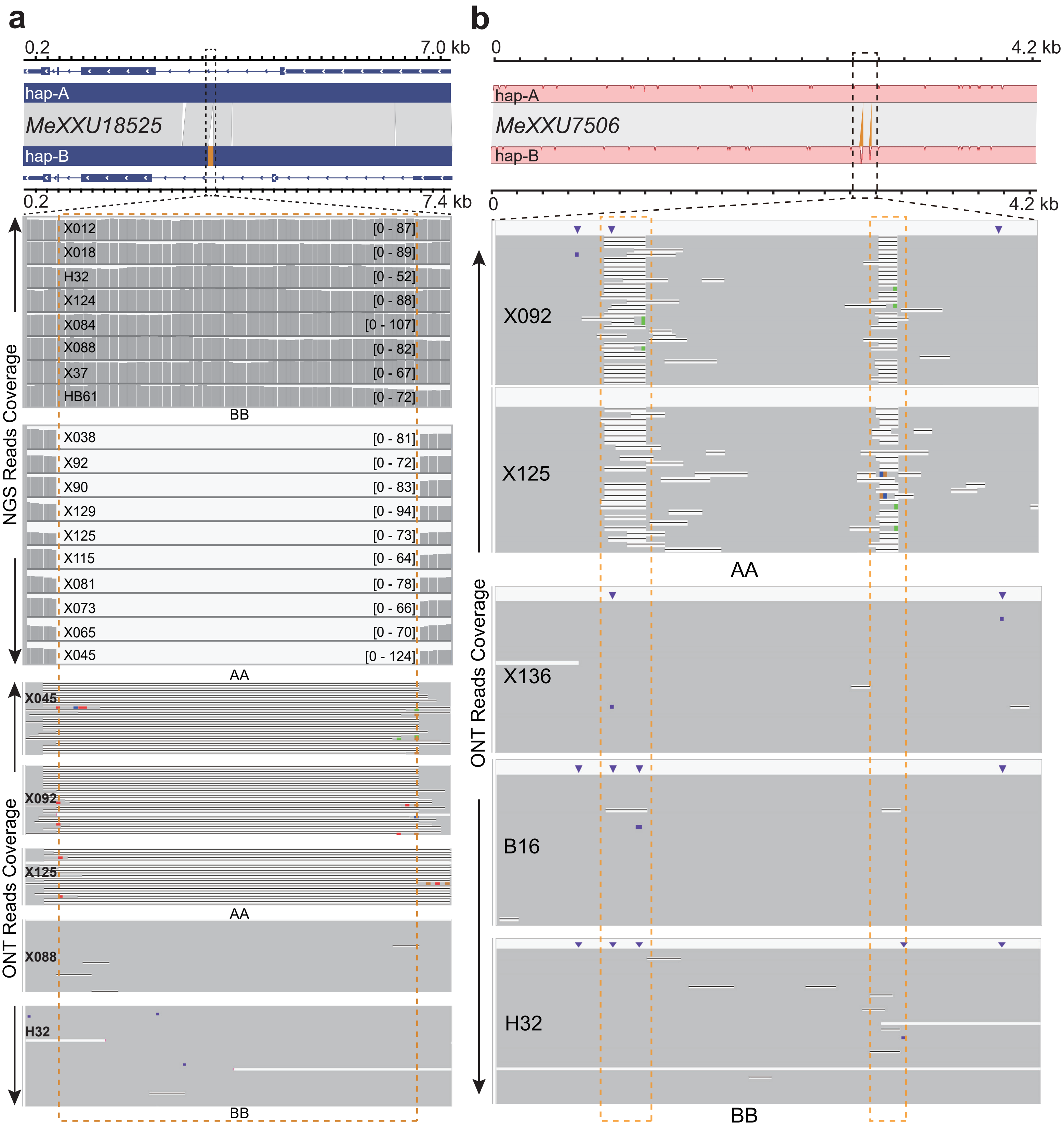

Fig. S15. Colinearity of *MeXXU18525* and *MeXXU7506* between haploid genomes and genotype identification in S_1 lines. (a) Colinearity of *MeXXU18525* between the two haploid genomes and reads coverage of the NGS and ONT sequencing data of the S_1 lines in the regions where structural variants exist in this gene, using the B haploid genome as a reference. (b) Colinearity of *MeXXU7506* between the two haploid genomes and reads coverage of the ONT sequencing data of the S_1 lines in the regions where structural variants exist in this gene, using the B haploid genome as a reference.

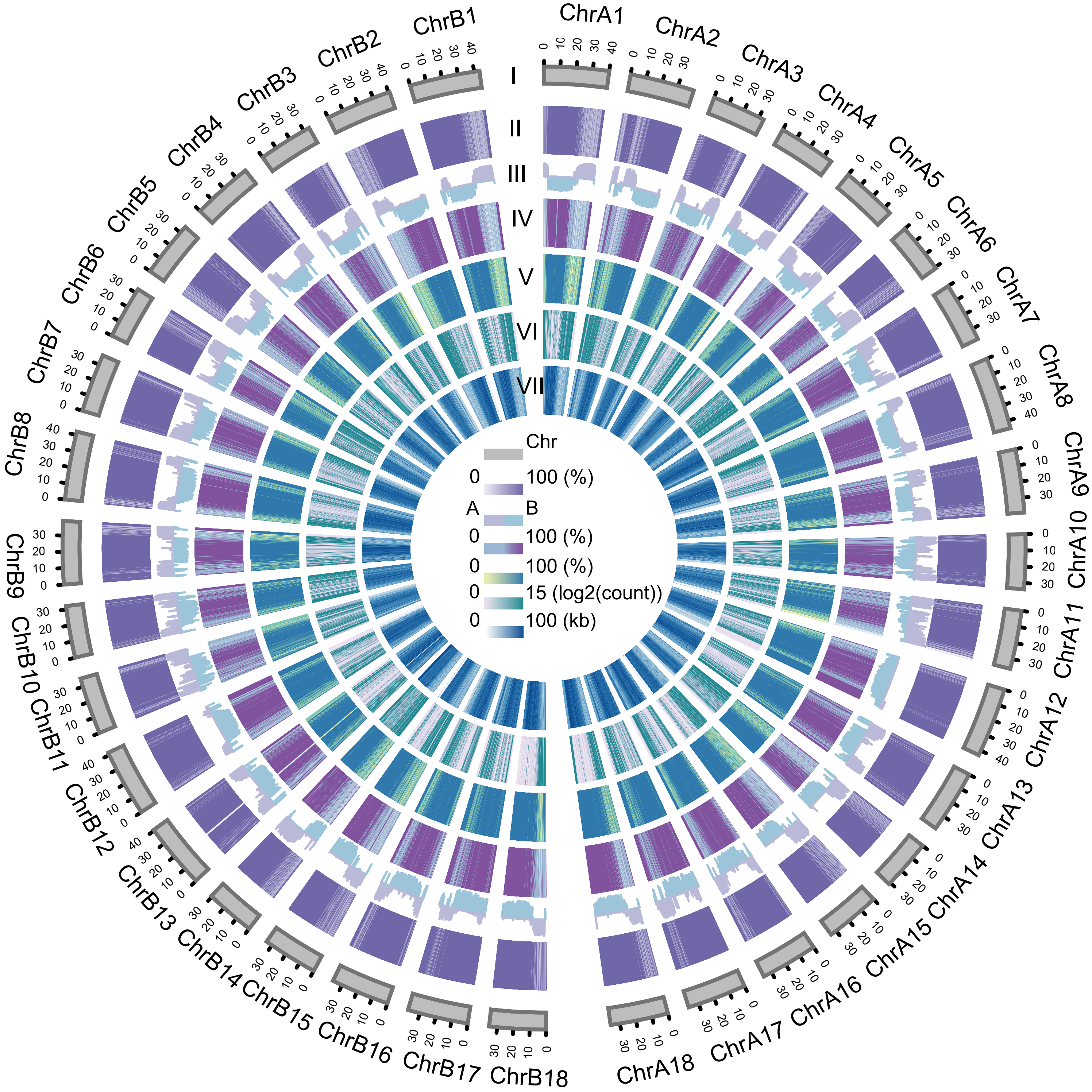

Fig. S16. The epigenetic landscape of the XX048 genome. (I) chromosomes, (II) CG methylation level, (III) A/B compartment, (IV) CHH methylation level, (V) CHG methylation level, (VI) gene expression level, (VII) TE proportion.

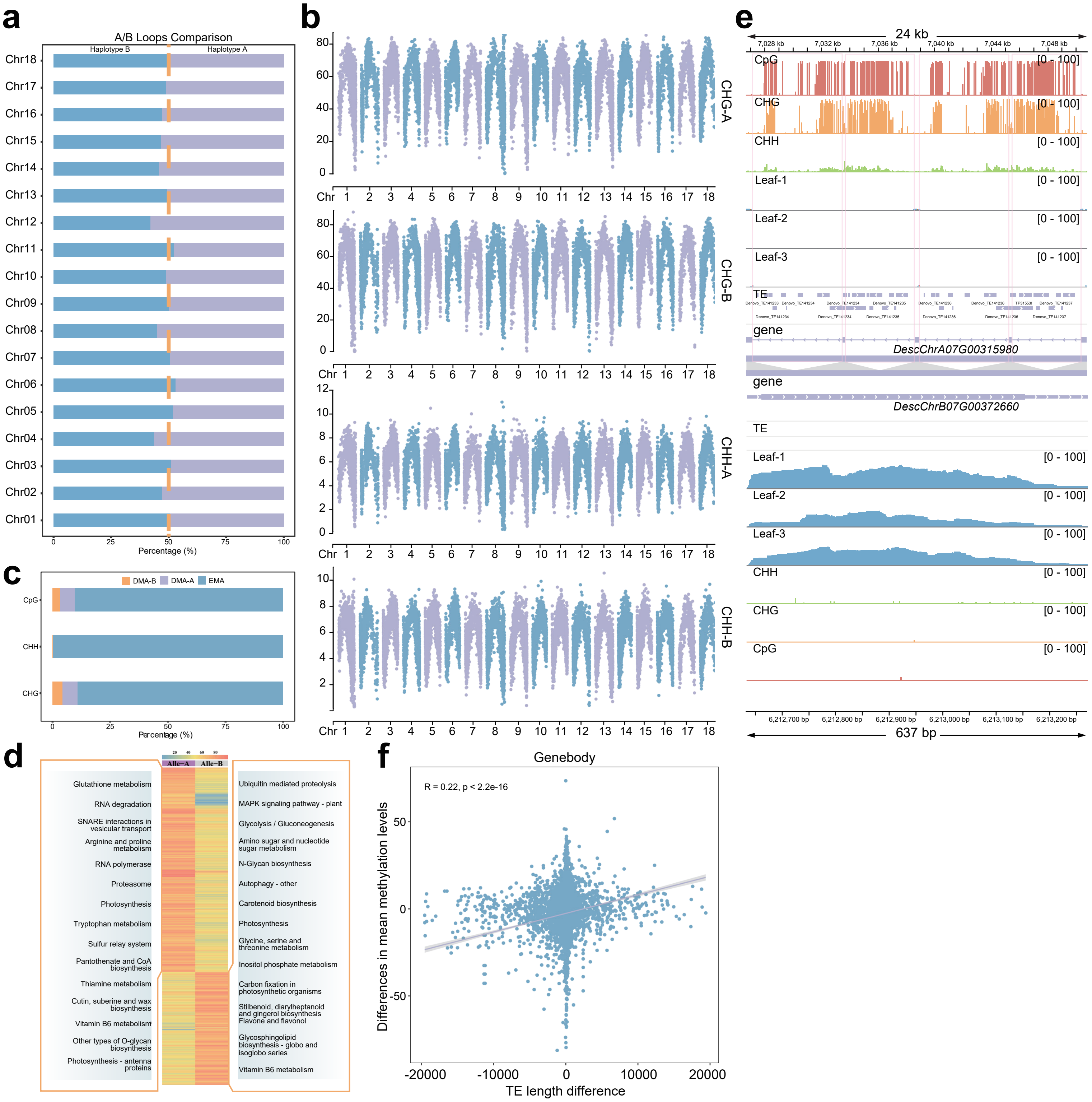

Fig. S17. Difference in epigenetic features between the two haploid genomes of XX048. (a) Different distribution of the number of loops between each pair of homologous chromosomes in the A and B haploid genome. (b) Distribution of allelic methylation levels with spatial location on each chromosome in the A and B haploid genome. (c) Stacked histogram of the proportion of differentially methylated alleles of CG, CHG, and CHH types in the alleles of the A and B haploid genomes. (d) KEGG enrichment analysis of differentially methylated alleles of CG types between A and B haploid genomes. (e) Visualization of other representative examples of TE insertions that result in SVs formation with varied methylation effects on allele expression levels using the IGV. (f) Pearson correlation test for TE length differences within 20 kb in genebody between A, B haploid genomic alleles and differences in CG methylation levels.

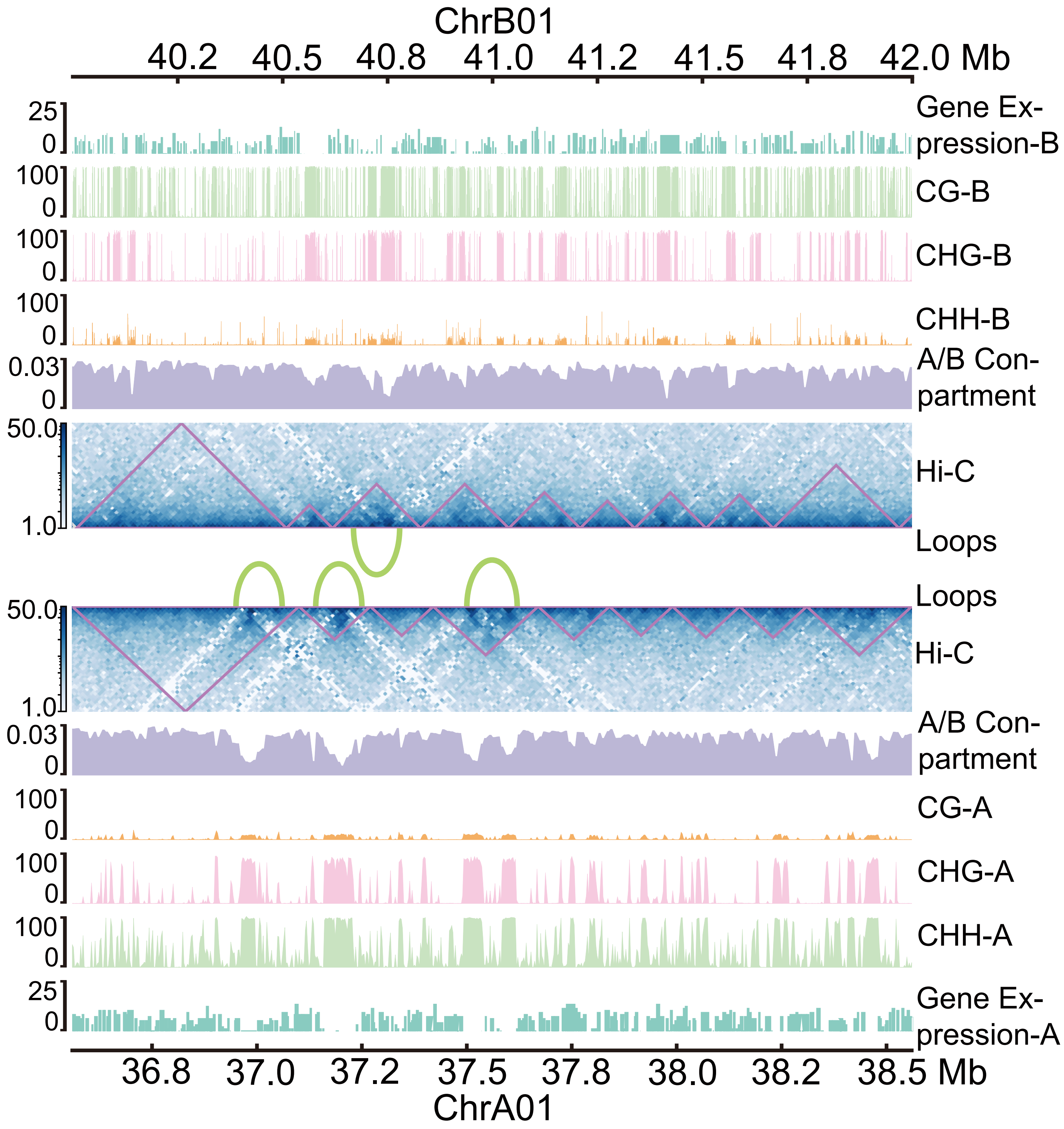

Fig. S18. Distribution of transcriptome and epigenome features in regions of high heterozygosity on homologous chromosome 1. Comparisons of the gene expression levels, CG, CHG, and CHH methylation levels, A/B compartment partitioning, Hi-C interaction matrices, TAD partitioning, and distribution of chromatin loops in the highly heterozygous region between the two haploid genomes.
