# Supplementary material for: Telomere-to-telomere assembly of cassava genome reveals the evolution of cassava and divergence of allelic expression
Source: Hortic Res. 2023 Oct 5;10(11):uhad200. doi: 10.1093/hr/uhad200 (PMC10673656; doi:10.1093/hr/uhad200)
Supplement: Supplementary_Methods_uhad200 [file supplementary_methods_uhad200.docx]

**T2T assembly of cassava genome reveals the evolution of cassava and divergence of allelic expression**

Xin-Dong Xu^1, #^, Ru-Peng Zhao^1, #^, Liang Xiao^2^, Liuying Lu^2^, Min Gao^1^, Yu-Hong Luo^1^, Zu-Wen Zhou^1^, Si-Ying Ye^1^, Yong-Qing Qian^1^, Bing-Liang Fan^1^, Xiaohong Shang^2^, Pingli Shi^2^, Wendan Zeng^2^, Sheng Cao^2^, Zhengdan Wu^2^, Huabing Yan^2, *^, Ling-Ling Chen^1, *^ and Jia-Ming Song^1, *^

**Supplementary Methods**

**Genomic DNA extraction**

Leaf tissue samples were procured from plants of the ‘Xinxuan 048’ variety. To conduct SMRT sequencing, high-molecular-weight DNA was procured employing CTAB technique, after which a standard SMRTbell library was developed, utilizing 50 μg of the extracted DNA, and the SMRTbell Express Template Prep Kit 2.0, following the manufacturer’s instructions (Pacific Biosciences, CA, USA).

**Illumina, Hi-C, ONT and HiFi sequencing**

To produce libraries for Illumina paired-end genome sequencing, we utilized the Truseq Nano DNA HT sample preparation kit and followed the standard protocol recommended by the manufacturer (Illumina). Subsequently, the Illumina HiSeq X Ten platform was utilized to sequence the produced libraries, generating 150-bp paired-end reads with a 350-bp insert size, providing an output of 84 Gb reads (∼249× coverage) for XX048. To generate a Hi-C library, we extracted *in-situ* cross-linked DNA from 700 ng of high-molecular-weight genomic DNA, and digested it with a restriction enzyme. Biotinylated cohesive ends of the digested genomic fragments were joined randomly to form chimeric junctions. Biotinylated DNA fragments were further enriched, and their size was reduced to 300-500bp during the sequencing library preparation step^1^. The Illumina HiSeq X Ten platform was used to perform the sequencing. Meanwhile, for ONT sequencing, we utilized the Oxford Nanopore 1D genomic DNA by ligation (SQK-LSK109) – PromethION (version GDE_9063_v109_revD_04Jun2018) protocol to prepare PromethION libraries, which were then sequenced on the Nanopore PromethION platform. Lastly, the PacBio Sequel II platform (Pacific Biosciences) was employed to sequence the genomes of XX048, producing 38 Gb HiFi reads (∼55× coverage).

**Genome assessment**

We utilized the quast (v5.0.2)^2^ to assess genome size and N50 length. Genome integrity was assessed using the BUSCO (v5.2.2)^3^ diploid database, while assembly continuity was evaluated using the LTR Assembly Index (LAI)^4,5^. Telomere searches were performed using the "TTTAGGG" sequence to determine the number of telomeres resulting from the assembly. The genome assembly was assessed using merqury (v1.3)^6^, which also enabled us to evaluate base accuracy.

**Repeat annotation**

For repeat annotation of the genome, we employed RepeatMasker (v4.0.9)^7^ based on the RepBase library (http://www.girinst.org/repbase). To build a *De novo* prediction library based on its sequence features, we used RepeatModeler (v1.0.11)^8^. We then used RepeatMasker (v4.0.9)^7^ to compare and predict duplicate sequences. Finally, we combined all duplicate prediction results and de-redunded them to obtain the final genomic duplicate sequence set.

**Gene annotation**

Exonerate (v2.4.0)^9^, Augustus (v3.3.2)^10^, Genscan (v1.0)^11^, and GlimmerHMM (v3.0.4)^12^ were used for de novo prediction of gene structure. RNA-seq data were reconstructed by stringtie (v2.1.4)^13^, and transcripts were obtained using TransDecoder (v5.1.0) (<https://github.com/TransDecoder/TransDecoder>) to predict the coding frames. MAKER (v2.31.10)^14^ was used to integrate the gene sets predicted by the various methods. After filtering, a non-redundant and more complete gene set is formed.

**Functional annotation**

There are two methods for gene function annotation, sequence similarity search (using Diamond BLASTP, parameter: -value (e-5)^15^). The KEGG^16^ annotation uses KOBAS (version: 3.0)^17^, a program that associates sequences with KEGG ORTHOLOGY and PATHWAY. The Uniprot^18^ database records the reciprocal correspondence between each protein family and the functional nodes of the genome^19^ that are used to predict the biological function of the protein sequences encoded by the genes. And pattern similarity search (This method uses InterProScan (version: v5.52-86.0)^20^ Search for CDD, Gene3D, Hamap, Panther, Pfam, Phobius, Pirsf, Pirsr, Prints, Prosite, Sfld and Sfld patterns in the second level database InterPro subdataset. In addition, comparing conserved sequences, motifs, and structural domains of the proteins with the InterPro, Prosite, Sfld, Smart, Superfamily, Tigrfam, and Tmhmm databases can provide further information. Structural domain prediction is performed using hmmscan (v3.3.2; parameter: e-value 0.01) (http://hmmer.org/) to identify conserved sequences, motifs, and structural domains of proteins.

**Noncoding RNAs annotation**

Using the structural features of tRNAs, we utilized tRNAscan-SE (v1.23)^21^ to identify tRNA sequences in the genome. For rRNA prediction, we employed the rRNA database. Additionally, to detect ncRNA sequences such as snRNA and miRNA in the genome, we used INFERNAL (v1.1.2)^22^ based on the Rfam database.

**Definition of alleles**

We defined alleles following the method published in the SC205 study, utilizing BLAST (v2.5.0+)^23^ and MCScanX^24^ to define the alleles. This approach enabled us to obtain a total of 22,839 allele pairs.

**Centromere prediction**

We utilized gene density, repetitive sequence density, and Hi-C interaction density maps to predict the potential location of mitophagy candidates. Furthermore, TRF (v4.09.1)^25^ was employed to identify potential mitophagy motif sequences within these regions, while gepard (v4.0.35)^26^ was utilized to compare the distribution of the predicted mitophagy motif sequences.

**Genotyping of progeny population**

For the genes we were particularly interested in, we performed fine genotyping of these genes using minimap (v2-2.26-r1175)^27^ and bwa (0.7.17-r1188)^28^, based on Illumina read coverage differences and ONT read mapping differences. After processing the comparison files using SAMtools (v1.15)^29^, we demonstrated reads coverage using IGV (v2.16.2)^30^ for some genes.

**Code availability**

The relevant code used in this study can be accessed for download through the link <https://cbi.gxu.edu.cn/download_files/cassava/Cassava_XX048/Code/>.

**References**

1. Xie, T. *et al.* De novo plant genome assembly based on chromatin interactions: a case study of Arabidopsis thaliana. *Mol. Plant* **8**, 489–492 (2015).

2. Gurevich, A., Saveliev, V., Vyahhi, N. & Tesler, G. QUAST: quality assessment tool for genome assemblies. *Bioinforma. Oxf. Engl.* **29**, 1072–1075 (2013).

3. Manni, M., Berkeley, M. R., Seppey, M., Simão, F. A. & Zdobnov, E. M. BUSCO Update: Novel and Streamlined Workflows along with Broader and Deeper Phylogenetic Coverage for Scoring of Eukaryotic, Prokaryotic, and Viral Genomes. *Mol. Biol. Evol.* **38**, 4647–4654 (2021).

4. Ou, S., Chen, J. & Jiang, N. Assessing genome assembly quality using the LTR Assembly Index (LAI). *Nucleic Acids Res.* **46**, e126 (2018).

5. Ou, S. & Jiang, N. LTR_retriever: A Highly Accurate and Sensitive Program for Identification of Long Terminal Repeat Retrotransposons. *Plant Physiol.* **176**, 1410–1422 (2018).

6. Rhie, A., Walenz, B. P., Koren, S. & Phillippy, A. M. Merqury: reference-free quality, completeness, and phasing assessment for genome assemblies. *Genome Biol.* **21**, 245 (2020).

7. Chen, N. Using RepeatMasker to identify repetitive elements in genomic sequences. *Curr. Protoc. Bioinforma.* **Chapter 4**, Unit 4.10 (2004).

8. Flynn, J. M. *et al.* RepeatModeler2 for automated genomic discovery of transposable element families. *Proc. Natl. Acad. Sci. U. S. A.* **117**, 9451–9457 (2020).

9. Slater, G. S. C. & Birney, E. Automated generation of heuristics for biological sequence comparison. *BMC Bioinformatics* **6**, 31 (2005).

10. Stanke, M., Diekhans, M., Baertsch, R. & Haussler, D. Using native and syntenically mapped cDNA alignments to improve de novo gene finding. *Bioinforma. Oxf. Engl.* **24**, 637–644 (2008).

11. Burge, C. B. Chapter 8 – Modeling dependencies in pre-mRNA splicing signals. *New Compr. Biochem.* **32**, 129–164 (1998).

12. Delcher, A. L., Bratke, K. A., Powers, E. C. & Salzberg, S. L. Identifying bacterial genes and endosymbiont DNA with Glimmer. *Bioinforma. Oxf. Engl.* **23**, 673–679 (2007).

13. Kovaka, S. *et al.* Transcriptome assembly from long-read RNA-seq alignments with StringTie2. *Genome Biol.* **20**, 278 (2019).

14. Holt, C. & Yandell, M. MAKER2: an annotation pipeline and genome-database management tool for second-generation genome projects. *BMC Bioinformatics* **12**, 491 (2011).

15. Buchfink, B., Xie, C. & Huson, D. H. Fast and sensitive protein alignment using DIAMOND. *Nat. Methods* **12**, 59–60 (2015).

16. Ogata, H. *et al.* KEGG: Kyoto Encyclopedia of Genes and Genomes. *Nucleic Acids Res.* **27**, 29–34 (1999).

17. Xie, C. *et al.* KOBAS 2.0: a web server for annotation and identification of enriched pathways and diseases. *Nucleic Acids Res.* **39**, W316-322 (2011).

18. UniProt Consortium, T. UniProt: the universal protein knowledgebase. *Nucleic Acids Res.* **46**, 2699 (2018).

19. Ashburner, M. *et al.* Gene ontology: tool for the unification of biology. The Gene Ontology Consortium. *Nat. Genet.* **25**, 25–29 (2000).

20. Blum, M. *et al.* The InterPro protein families and domains database: 20 years on. *Nucleic Acids Res.* **49**, D344–D354 (2021).

21. Chan, P. P., Lin, B. Y., Mak, A. J. & Lowe, T. M. tRNAscan-SE 2.0: improved detection and functional classification of transfer RNA genes. *Nucleic Acids Res.* **49**, 9077–9096 (2021).

22. Nawrocki, E. P. & Eddy, S. R. Infernal 1.1: 100-fold faster RNA homology searches. *Bioinforma. Oxf. Engl.* **29**, 2933–2935 (2013).

23. Boratyn, G. M. *et al.* Domain enhanced lookup time accelerated BLAST. *Biol. Direct* **7**, 12 (2012).

24. Wang, Y., Li, J. & Paterson, A. H. MCScanX-transposed: detecting transposed gene duplications based on multiple colinearity scans. *Bioinforma. Oxf. Engl.* **29**, 1458–1460 (2013).

25. Benson, G. Tandem repeats finder: a program to analyze DNA sequences. *Nucleic Acids Res.* **27**, 573–580 (1999).

26. Krumsiek, J., Arnold, R. & Rattei, T. Gepard: a rapid and sensitive tool for creating dotplots on genome scale. *Bioinforma. Oxf. Engl.* **23**, 1026–1028 (2007).

27. Li, H. Minimap2: pairwise alignment for nucleotide sequences. *Bioinforma. Oxf. Engl.* **34**, 3094–3100 (2018).

28. Li, H. & Durbin, R. Fast and accurate short read alignment with Burrows-Wheeler transform. *Bioinforma. Oxf. Engl.* **25**, 1754–1760 (2009).

29. Li, H. *et al.* The Sequence Alignment/Map format and SAMtools. *Bioinforma. Oxf. Engl.* **25**, 2078–2079 (2009).

30. Robinson, J. T., Thorvaldsdottir, H., Turner, D. & Mesirov, J. P. igv.js: an embeddable JavaScript implementation of the Integrative Genomics Viewer (IGV). *Bioinforma. Oxf. Engl.* **39**, btac830 (2023).
